# Behaviour change techniques reported in intervention studies of alcohol and tobacco use: a rapid review

**DOI:** 10.1080/21642850.2025.2554182

**Published:** 2025-09-23

**Authors:** Anuijan Chandran, Scott Veldhuizen, Kamna Mehra, Terri Rodak, Danial Vagharfard, Michelle Pham, Laurie Zawertailo, Jurgen Rehm, Christian S. Hendershot, Peter Selby, Nadia Minian

**Affiliations:** a INTREPID Lab, Centre for Addiction and Mental Health, Toronto, ON, Canada; b Institute of Medical Science, Temerty Faculty of Medicine, University of Toronto, Toronto, Canada; c Department of Family and Community Medicine, University of Toronto, Toronto, ON, Canada; d Campbell Family Mental Health Research Institute, Centre for Addiction and Mental Health, Toronto, ON, Canada; e Department of Psychiatry, University of Toronto, Toronto, ON, Canada; f Dalla Lana School of Public Health, University of Toronto, Toronto, ON, Canada; g Department of Pharmacology and Toxicology, University of Toronto, Toronto, ON, Canada; h Institute for Mental Health Policy Research, Centre for Addiction and Mental Health, Toronto, ON, Canada; i Department of Population and Public Health Sciences and Institute for Addiction Science, Keck School of Medicine of the University of Southern California, Los Angeles, CA, USA; j CAMH Mental Health Sciences Library, Department of Education, Centre for Addiction and Mental Health, Toronto, ON, Canada

**Keywords:** Review, alcohol, tobacco, reduction, cessation, behaviour change techniques

## Abstract

**Background:**

Clinical guidelines recommend addressing alcohol and tobacco use simultaneously, but few providers offer brief alcohol interventions routinely, and these behaviours are often treated separately. While several interventions targeted dual use, there remains a gap in identifying behaviour change techniques (BCTs) designed to modify processes controlling dual use.

**Objective:**

To identify commonly used BCTs in interventions targeting both alcohol and tobacco use, their modes of delivery, and explore which BCTs are associated with smoking cessation and/or alcohol reduction.

**Methods:**

Following Cochrane recommendations, a rapid review to identify BCTs showing promise for reducing dual use was conducted. Using an eligibility criteria, we retrieved relevant papers from databases and used the Behavioural Change Taxonomy V1 tool to identify BCTs showing promise.

**Results:**

Thirty-eight articles of the initial systematic search of 2987 papers met the criteria for full article review. Goal setting, action planning, and pharmacological support were the most common BCTs identified. Most studies (33, 87%) had a low or moderate risk of bias. Of these 33 studies, 13 studies (39%) reported statistically significant outcomes of reduction or cessation in smoking behaviour and alcohol consumption. Face to face (25, 76%) was the most common intervention delivery method.

**Conclusion:**

Clinical trials identify goal setting, action planning and problem solving to address the dual use of tobacco and alcohol. Systematic reviews and meta-analyses are needed to evaluate the true impact of these programmes. Future studies should minimally include these BCTs and study the interactional effects of these BCTs on the efficacy of the intervention.

## Introduction

Tobacco and alcohol use are leading contributors to preventable disease, disability, and death worldwide (WHO Report on the Global Tobacco Epidemic, 2023; World Health Organization; World Health Organization, 2018). Collectively, these substances account for over 11 million deaths annually, representing a substantial share of the global burden of non-communicable diseases such as cancer, cardiovascular and respiratory diseases, and liver cirrhosis (WHO Report on the Global Tobacco Epidemic, 2023; World Health Organization; World Health Organization, 2018). When used together, their effects are not merely additive but synergistic, significantly increasing the risk of diseases such as aero-digestive cancers and exacerbating mental health conditions (Hashibe et al., 2007; Pelucchi et al., 2008).

Beyond the immense health toll, the economic and societal costs of alcohol and tobacco use are substantial. In Canada alone, these two substances together accounted for over $31 billion in direct and indirect costs in 2020—$19.7 billion for alcohol and $11.5 billion for tobacco—due to healthcare utilisation, lost productivity, criminal justice involvement, and other social consequences (Canadian Centre on Substance Use and Addiction). At a societal level, alcohol and tobacco contribute to increased hospital admissions, reduced workplace productivity, family disruption, and impaired quality of life, particularly among marginalised populations (Peter, 2011; Rhem, 2006). These burdens highlight the need for effective, scalable, and targeted interventions that reduce both alcohol and tobacco consumption.

Despite often being addressed separately in public health and clinical practice, alcohol and tobacco use frequently co-occur and interact in ways that complicate treatment and prevention efforts. Individuals who consume alcohol are more likely to smoke, and heavier alcohol use is associated with higher rates of nicotine dependence (Statistics Canada; Zvolensky et al., 2015). Additionally, alcohol consumption undermines smoking cessation by increasing cravings and relapse risk (Kahler et al., 2009). In Canada, a high proportion of individuals with Alcohol Use Disorder (AUD) also meet the criteria for Tobacco Use Disorder (TUD), making concurrent treatment both necessary and potentially more effective (Prochaska et al., 2004). As such, integrated interventions that simultaneously target alcohol and tobacco use are essential to reduce overlapping health harms and to improve both individual and public health outcomes.

Best-practice guidelines recommend that alcohol and tobacco use be addressed concurrently, given their frequent co-occurrence and interactive effects on health outcomes (Greenhalgh & Papoutsi, 2018; Hacker & Kang, 2021; Loheswaran et al., 2015; Tait et al., 2019). Interventions designed to target both behaviours simultaneously vary widely in their effectiveness (Bully et al., 2015; Cooney et al., 2015). This variability makes it difficult to identify the active components responsible for behaviour change, posing a challenge for anyone seeking to adopt or replicate evidence-based approaches to treating co-use of alcohol and tobacco (Greenhalgh & Papoutsi, 2018).

One promising approach to overcoming this challenge is the use of the Behaviour Change Technique Taxonomy version 1 (BCTTv1), a standardised framework that identifies and categorises the components of behavioural interventions. Behaviour change techniques (BCTs) are defined as observable, replicable, and irreducible elements intended to alter behavioural processes (Michaelsen & Esch, 2022; Michie et al., 2013). The BCTTv1 comprises 93 distinct BCTs organised into 16 hierarchical groups and is widely used to improve transparency and replicability in intervention research.

Evidence from systematic reviews in the alcohol and tobacco domains suggests that interventions with multiple BCTs tend to be more effective than those with fewer components (Carraça et al., 2021). In particular, techniques such as goal setting, self-monitoring, and providing feedback—especially when delivered via tailored digital platforms like mobile apps or SMS—have been associated with improved engagement and outcomes (Melo et al., 2025; Trumpf et al., 2023). These shared features offer promising directions for designing synergistic interventions, but to date, no systematic review has focused specifically on identifying the active ingredients of integrated alcohol and tobacco interventions.

This gap presents a significant barrier to both intervention development and public health impact. Without a clearer understanding of the specific BCTs that contribute to success in addressing co-use, efforts to replicate or adapt effective programmes are limited. To address this need, the present review aims to identify and synthesise the BCTs used in interventions targeting both tobacco and alcohol use, examine their modes of delivery, and explore associations with behaviour change outcomes such as smoking cessation and alcohol reduction.

## Methods

### Ethics statement

Since this study is a rapid systematic review of existing literature and does not involve the collection of primary data or interaction with human or animal subjects, ethical approval was not required. Informed consent was not obtained as the research relies solely on publicly available, published studies.

A rapid review methodology was chosen, because it supports efficient and timely review while also being cost-effective compared to traditional systematic reviews (Tricco et al., 2015). The rapid review was conducted according to Cochrane recommendations (Cochrane) and following the Preferred Reporting Items for Systematic Reviews and Meta-Analyses (PRISMA) guidelines (Liberati et al., 2009). The study was registered with PROSPERO, an international prospective systematic review register (CRD42023445492).

### Eligibility criteria

To determine study eligibility, we used PICOD criteria: **P**opulation: Adults who use both tobacco and alcohol. **I**ntervention: Patient-level behavioural interventions for dual use of alcohol and tobacco. **C**omparator: Any control group (e.g. treatment as usual, addressing only one substance (tobacco or alcohol), consecutive treatment). **O**utcome: Objective and self-report measures of tobacco and alcohol use. **D**esign: Quantitative designs (e.g. randomised control trial, pre–post designs). The inclusion criteria consisted of (1) studies involving interventions provided for both alcohol and tobacco use; (2) published in English; (3) inclusive of adults (18 years and above); (4) with patient-level behavioural interventions; (5) only clinical trials with control groups; (6) quantitative and/or mixed methods study design; and (7) primary research studies.

The exclusion criteria consisted of (1) intervention focused solely on alcohol use or tobacco use; (2) studies not published in English; (3) study population only consisting of adolescents; (4) intervention provided for vaping/e-cigarettes only; (5) observational and qualitative study designs; (6) outcomes did not include either objective or self-report measures of tobacco and alcohol use; (7) publications that were theses, conference proceedings, protocols, reviews, books, and grey literature. (8) For the analysis, studies with a high risk of bias were also excluded.

### Information sources and search strategy

In collaboration with the research team, a health sciences librarian (TR) developed, tested, refined, and finalised a core search strategy in MEDLINE, which was then translated for use in additional databases. Searches were conducted in the following four databases on July 10, 2023 and again on June 6, 2024: MEDLINE (Ovid), Embase (Ovid), APA PsycInfo (Ovid), and CINAHL (EBSCO). All strategies used database-specific subject headings, keywords in natural language, and advanced search operators (especially proximity operators) to capture three concepts: Tobacco use or cessation, alcohol use or cessation, and treatment interventions. The strategy design took a multi-stranded approach, which was necessary to capture the myriad ways in which concurrent tobacco and alcohol use is described in the literature. Animal studies and articles on adolescents only were filtered out via subject headings, and a search filter from Canada’s Drug Agency was used to limit to clinical trials only (Canada’s Drug Agency). An additional string identified systematic or scoping reviews to be used for handsearching their reference lists. No year or language limits were applied. Conference abstracts and preprints were removed when possible. Full strategies for all databases can be found in (Appendix A).

### Selection process

After conducting the initial literature search, all search results were imported into a reference manager and then into the systematic review software Covidence (Veritas Health Innovation), for screening by 5 reviewers (AC, MP, DV, KM, NM). The screening process adhered to the eligibility criteria outlined previously, specifically focusing on studies involving patient-level behavioural interventions targeting both alcohol and tobacco use in adults.

To ensure consistency in applying the inclusion and exclusion criteria, four reviewers (AC, MP, DV, KM) participated in a pilot screening to calibrate the forms used during title and abstract screening, full-text screening, data extraction, and quality assessment, as well as to ensure consistency and resolve any discrepancies. For the pilot screening, all reviewers conducted title and abstract screening on 20% (n = 462) of the studies and full-text screening on 20% (n = 20) of the selected studies that were included in the title and abstract screening stage. Conflicts were resolved through meetings and reaching consensus through discussion or by a third reviewer (NM).

During the pilot title and abstract screening, there were 129 (28%) conflicts on either excluding or including an article. Due to the high number of conflicts, we conducted another round of title and abstract screening (*n* = 239) articles to ensure consistency and following the inclusion and exclusion criteria. After the additional round, we only had 2 (0.84%) conflicts.

After the pilot screening, one reviewer (AC) screened the title and abstracts of the remaining 1375 articles. A total of 93 articles moved onto the full-text screening. The other reviewers verified the resulting exclusion list of 1983 articles from the title and abstract screening to ensure that eligible studies were not missed. Similarly, after the pilot screening of the full-text articles, one reviewer (AC) reviewed 72 studies, and the excluded list of 55 articles were reviewed by other reviewers. Conflicts were resolved through meetings and reaching consensus through discussion or by a third reviewer (NM).

Overall, the selection process was designed to ensure that studies meeting the eligibility criteria were identified and considered for review. Regular meetings and discussions during the screening stages helped ensure consistency, and using a third reviewer to resolve conflicts added reliability to the process.

### Data extraction

A data extraction form was developed based on the goals of the review and literature about BCTs (Michie et al., 2013). This included: author name, year of publication, country where the study was conducted, study design, study population, demographics of study population, sample size, measures, intervention, outcomes. For each outcome (alcohol reduction/ smoking cessation), the intervention effect was extracted and categorised as reduction or no reduction.

Type of BCTs used was defined using the BCT V1 taxonomy tool (BCT Taxonomy (v1)). The BCTs extracted included goals and planning (BCT 1), feedback and monitoring (BCT 2), social support (BCT 3), shaping knowledge (BCT 4), natural consequences (BCT 5), comparison of behaviour (BCT 6), associations (BCT 7), repetition and substitution (BCT 8), comparison of outcomes (BCT 9), reward and threat (BCT 10), regulation (BCT 11), antecedents (BCT 12), scheduled consequences (BCT 14), and self-belief (BCT 15).

BCTs delivery format was extracted using the mode of delivery ontology (Marques et al., 2020) (i.e. Face to face, over the phone, web, app, text messages, mass media and other). Some modes of delivery (over the phone, web, app, text messages) were grouped together as digital technologies. For studies where the mode of delivery was not explicitly mentioned, we inferred this information based on the contextual details provided. For interventions that included multiple BCTs, we noted the types of BCTs used and the number of studies that used each combination.

All reviewers conducted a pilot data extraction of 9 studies (20%). During the pilot extraction, the reviewers compared and discussed the extraction results to improve the accuracy and comparability of reviewers. The remaining 29 studies were extracted by one reviewer individually (AC), and the other reviewers verified the accuracy and completeness of the data extracted; all issues were addressed as a team during regular study meetings.

### Quality assessment

To assess the level of bias in each of the included studies, we used the Joanna Briggs Institute’s (JBI) critical appraisal tool to assess the quality of the randomised controlled trials (RCTs) and case–control studies (Joanna Briggs Institute, 2024). The RCT appraisal tool consisted of 13 questions and the case–control critical appraisal tool of 10 questions. To determine the level of bias in the studies, the percentage of ‘Yes’ answers on the quality assessment tool was calculated; if questions were not applicable, they were removed from the denominator. Studies were deemed low risk of bias if they scored 70% or above, moderate risk if they scored between 41% and 69% and high risk if they scored 40% or lower. The same pilot process for data extraction was conducted for quality assessment and it was conducted through Covidence.

### Data synthesis

The data synthesis aimed to identify BCTs commonly used in interventions targeting tobacco and alcohol consumption, with the goal of advancing the design of integrated interventions for these behaviours. We used narrative synthesis (Baradaran et al., 2016) to summarise the extracted information. All reviewers were trained in coding using the BCTTv1 using the online training (BCT Taxonomy v1 Online Training). To determine which BCTs were associated with the desired outcomes—smoking cessation and/or alcohol reduction—we classified the interventions into two categories: ‘showing promise’ or those ‘lacking sufficient evidence’. An intervention was categorised as ‘showing promise’ if it demonstrated statistically significant improvements in smoking cessation and alcohol reduction, smoking cessation alone, or alcohol reduction alone compared to the control group or baseline measures. Conversely, interventions were labelled ‘lacking sufficient evidence’ if no statistically significant improvements were observed. To enhance the validity of our synthesis, we prioritised findings from studies with robust methodological quality by including only those assessed as having a medium or low risk of bias, a practice endorsed by others (Katikireddi et al., 2015; Wood et al., 2008). We created tables (Appendix B) summarising how often different BCTs were used, the relationship between BCTs and outcomes, and a summary of outcomes and how the interventions were delivered. This was done to find any consistent patterns related to the types of BCTs that were linked to effective interventions.

## Results

**Figure 1.**
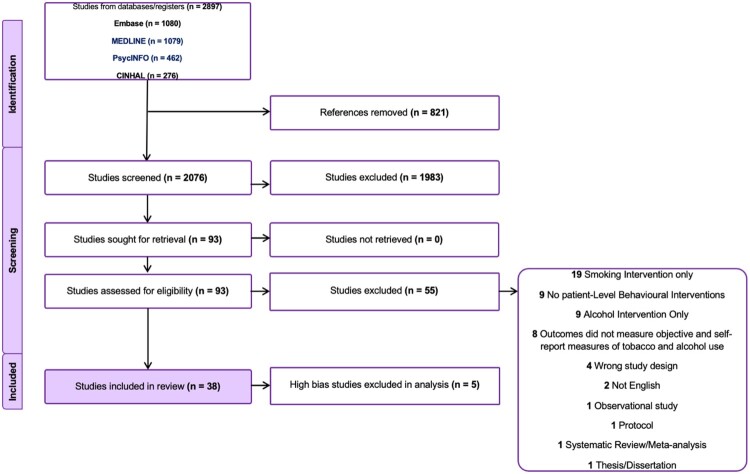
PRISMA flow diagram detailing the number of studies included and excluded in the review.

We screened 2076 studies during the title and abstract screening, 93 studies during the full-text screening and 38 studies were finalised for data extraction and quality assessment (Figure 1).

### Study characteristics

Most of the studies included in this review were RCTs (n = 33), with the remaining being case–control studies (n = 5). Studies were conducted in 12 different countries, with the largest number in the United States (n = 18, 47%) followed by the United Kingdom (n = 5, 13%).

Seventeen studies (45%) focused on interventions targeting the general population of adults who were currently smoking and using alcohol, 7 recruited people with upcoming surgeries or treatments (18%) and 4 recruited students (11%).

Most of the studies (n = 26) had a moderate risk of bias, 7 low risk of bias and 5 a high risk of bias. The 5 studies deemed high bias were excluded from the analysis and are only included in Table B1, which describes the summary of the studies included in this review as well as the risk of bias.

### Behaviour change techniques

A total of 27 BCTs were identified across all articles. The most common BCT used was goal setting (BCT 1.1) (n = 21, 64%), followed by action planning (BCT 1.4) (n = 17, 52%), pharmacological support (these interventions happened in the context of other behavioural interventions) (BCT 11.1) (n = 15, 45%), problem–solving (BCT 1.2) (n = 15, 45%), and information about health consequences (BCT 5.1) (n = 12, 36%).

Goal setting (BCT 1.1) was operationalised by setting reduction goals and/or quit dates. Action planning (BCT 1.4) involves helping develop strategies to cope with cravings. Pharmacological support (BCT 11.1) meant providing or encouraging the use of pharmacotherapies (Nicotine Replacement Therapy (NRT), Varenicline, Bupropion and/or Naltrexone) to change behaviour. Problem-solving (BCT 1.2) included identifying situations where cravings are experienced. Information about health consequences (BCT 5.1) meant information about the harmful effects of hazardous alcohol and smoking use and the benefits of reducing or stopping consumption.

The number of BCT categories used in a single study ranged from 2 to 9. Thirteen studies used both goal setting (BCT 1.1) and action planning (BCT 1.4) and 12 studies used both goal setting (BCT 1.1) and problem solving (BCT 1.2). Table B2 lists all the BCTs identified in the low or moderate bias studies.

### Association between individual BCTs and smoking cessation and/or alcohol reduction

Among the 33 studies reviewed, 13 demonstrated a significant reduction in both alcohol reduction and smoking. Two demonstrated significant smoking cessation, but not alcohol reduction. One demonstrated significant alcohol reduction and 17 did not demonstrate significant alcohol and/or tobacco reduction. Detailed outcomes for each study can be found in Table B1.

Thirty-nine percent of the studies (13 of 33) were deemed as ‘showing promise’. Out of these studies, 69% (9 out of 13) used goal setting (BCT 1.1) 27% (8 out of 13) used pharmacological support (BCT 11.1) and 46% (6 out of 13) used action planning (BCT 1.4). All studies that demonstrated reductions in both alcohol and tobacco use included at least two BCTs. Fifty-two percent of the studies (17 of 33) were deemed as ‘lacking sufficient evidence’. Out of these studies, 53% (9 out of 17) used goal setting (BCT 1.1), 53% (9 out of 17) used action planning (BCT 1.4), and 47% (8 out of 17) used problem solving (BCT 1.2). Similar BCTs are seen when comparing the studies that showed promise and lacked sufficient evidence. This indicates that contextual factors, such as the method of delivery and characteristics of participants may play a role in determining the effectiveness of the intervention rather than implementing the BCTs alone. The summarised BCT activity of the statistically significant studies for alcohol and tobacco reduction, alcohol-only reduction, and tobacco-only reduction are listed in Table B3.

### Association between mode of delivery and smoking cessation and/or alcohol reduction

Out of the 33 studies we analysed, 25 delivered their intervention face to face, 14 studies used digital technologies to deliver their intervention (10 over the phone; 4 using a website; 2 using SMS; and 1 using an app). Six out of 14 studies delivered the digital intervention alongside the face-to-face intervention. Among the 25 studies that delivered their interventions face-to-face, 11 demonstrated statistically significant reductions in both alcohol and tobacco use, 2 showed reductions in tobacco use only, and 12 did not show any significant reductions.

Of the 25 interventions delivered face-to-face that resulted in reductions in both alcohol and tobacco use, the most common BCT used was goal setting (BCT 1.1) (n = 7), followed by action planning (BCT 1.4) (n = 5) and problem-solving (BCT 1.2) (n = 5).

Out of the 14 studies that delivered interventions digitally, 6 demonstrated statistically significant reductions in both alcohol and tobacco use, 1 showed reductions in alcohol use only, and 8 did not show any significant reductions. The most common BCT used resulting in reductions in both alcohol and tobacco use was goal setting (BCT 1.1) (n = 6), followed by action planning (BCT 1.4) (n = 4), information on health consequences (BCT 5.1) (n = 4), problem-solving (BCT 1.2) (n = 3), and pharmacological support (BCT 11.4) (n = 1). The summarised outcomes and modes of delivery of the studies are listed in Table B4.

## Discussion

This rapid review aimed to identify the most common BCTs used in interventions targeting the dual use of alcohol and tobacco, as well as their modes of delivery. While 38 studies met our inclusion criteria, five were excluded from the analysis as they were high risk of bias. The majority of the studies were RCTs, with a smaller number of case–control studies. Although several reviews have examined BCTs in smoking cessation interventions (Bartlett et al., 2014; Black et al., 2020; McCrabb et al., 2019) and alcohol interventions (Fergie et al., 2018; Humphreys et al., 2021; Sorcher & Branscum, 2022), this review specifically focused on interventions addressing both alcohol and tobacco use.

Of the 93 discrete BCTs defined in the BCT taxonomy, only 27 were used in the studies we examined. This aligns with other reviews, which have found that only a small subset of BCTs is commonly applied (Bartlett et al., 2014; Black et al., 2020; Fergie et al., 2018; Humphreys et al., 2021; McCrabb et al., 2019; Sorcher & Branscum, 2022). This limited use could stem from a combination of practical constraints, researchers’ familiarity, and the alignment of certain techniques with the specific aims of the interventions. Intervention designers might gravitate toward a smaller set of well-established BCTs that have been widely studied, tested, and shown to be effective. Additionally, some BCTs, such as adding objects to the environment (BCT 12.5), or remove aversive stimulus (BCT 7.5) may be more complex to implement or harder to operationalise within the constraints of a programme or study. Techniques that require more time, resources, or specialised skills, such as associative learning (BCT 7.8) or material incentives/ rewards (10.1 or 10.2), may deter researchers, particularly when resources are limited.

Similar to other reviews on behaviour change interventions (Bartlett et al., 2014; Black et al., 2020; Fergie et al., 2018; Humphreys et al., 2021; McCrabb et al., 2019; Sorcher & Branscum, 2022), this review found that interventions targeting both smoking cessation and alcohol use, tested in high-quality trials, employed an average of four BCTs, with a range of 2 to 9. In addition, all studies showing reductions in both alcohol and tobacco use incorporated two or more BCTs. This finding aligns with evidence from Michie and colleagues, who have emphasised that combining multiple BCTs can enhance intervention effectiveness. The identified BCTs for alcohol and smoking are similar to those used in smoking cessation interventions alone. This suggests that many BCTs effective for smoking may also apply to alcohol, which highlights the behaviour change mechanisms used for both alcohol and tobacco are common. While the studies varied in population, country, and intervention design, they consistently demonstrated the importance of specific BCTs, particularly goal setting (BCT 1.1), action planning (BCT 1.4), pharmacological support (BCT 11.1), and information on health consequences (BCT 5.1) in facilitating reductions in alcohol and tobacco consumption.

Goal setting (BCT 1.1) was the most frequently used BCT across the studies, used in 21 interventions. Eighty-one percent (17 of 21) of the interventions that used goal setting demonstrated significant improvements in these behaviours. Also, in over 65% of the studies (17 out of 26) that reported significant reductions in alcohol use, tobacco use, or both, goal setting played a central role. These results align with existing literature, which has long recognised goal setting as a highly effective method for promoting behaviour change in both smoking cessation and alcohol reduction efforts. Goal-setting theory (Jeong et al., 2023) postulates that clearly defined and challenging goals can significantly enhance motivation and performance by providing direction and purpose (Locke & Latham, 2002). In the context of the reviewed studies, goal setting was often operationalised by encouraging participants to set reduction goals or establish quit dates. By defining clear targets, participants could direct their efforts and maintain motivation, which may explain why studies employing goal setting saw significant reductions in both alcohol and tobacco use.

Action planning (BCT 1.4), which involves developing strategies to cope with cravings and potential triggers, was also frequently associated with successful outcomes. Of the 17 studies that used ‘action planning’, 12 (70%) showed significant reductions in smoking and/or alcohol. Action planning was used in 46% of the studies that reported significant reductions in alcohol use, tobacco use, or both. Its frequent association with positive outcomes may be due to its focus on helping individuals anticipate future challenges and having thought of healthy responses in advance, thus helping participants become better equipped to handle high-risk situations, without restoring to previous unhealthy/harmful behaviours. Action planning was mostly delivered in digital interventions which may further enhance its effectiveness by providing timely support and personalised strategies in real-world settings. For example, a study examining the efficacy of action planning in a smoking cessation intervention found a positive association with continued abstinence, suggesting that structured planning helps individuals maintain behaviour changes over time (Bolman et al., 2015).

Pharmacological support (BCT 11.4), which typically involved providing or encouraging the use of pharmacotherapies (which include NRT, Varenicline, Bupropion and/or Naltrexone) to change behaviour was another commonly used BCT, used in 15 studies. Previous reviews have similarly emphasised the value of including pharmacological supports to maximise the likelihood of success in smoking cessation and alcohol reduction. In our review, pharmacological support was often delivered alongside other BCTs like goal setting and action planning, which likely enhanced its effectiveness by addressing both the physiological and behavioural aspects of addiction. This combined approach has been shown to increase the likelihood of success. While the pharmacological component can help reduce cravings and withdrawal symptoms, the behavioural strategies provide individuals with the skills needed to navigate everyday triggers and challenges. The consistent use of this combination in the studies reviewed aligns with the evidence that integrating pharmacological and behavioural approaches is an effective way to promote smoking cessation and alcohol reduction (Lindson et al., 2023; Wood et al., 2023).

Twenty-five interventions in our review were delivered face-to-face, 11 of which reported reductions in both alcohol and tobacco use (44%). Face-to-face interventions may be effective by fostering a strong therapeutic relationship, creating a sense of accountability, and increasing motivation. These findings are consistent with previous research that has shown the benefits of face-to-face delivery, particularly for complex behaviour change interventions (Meyer-Schwickerath et al., 2022).

In our review, we found that digital interventions—delivered via phone, website, SMS, or apps—were commonly utilised. Of the 14 studies incorporating these digital methods, six (43%) reported reductions in both alcohol and tobacco use. This aligns with findings from other reviews that highlight the effectiveness of delivering alcohol interventions and smoking cessation interventions digitally (Griffiths et al., 2018; Nair et al., 2015; Oh et al., 2022; Sha et al., 2022). The success of these interventions may be attributed to several advantages they offer, such as ongoing support through automated systems and the facilitation of self-monitoring and feedback, both of which are crucial for sustaining behaviour change (Griffiths et al., 2018; Nair et al., 2015; Oh et al., 2022; Sha et al., 2022). However, given the wide variety of digital formats, interpreting the results is challenging. The relative scarcity of studies focusing on specific digital methods underscores the need for further exploration, especially as technology continues to evolve and becomes more integrated into healthcare delivery.

### Strengths and limitations

This review has several strengths. First, it comprehensively synthesises findings from a diverse set of RCTs and case–control studies across different countries and populations, providing a broad overview of how BCTs are applied in interventions that address both alcohol and tobacco use. Using the BCTTv1 taxonomy, we were able to categorise and compare the BCTs employed in each study, which enhances the reliability of our findings and allows for a systematic analysis of effective techniques. Additionally, the inclusion of studies with both face-to-face and digital intervention methods enabled us to examine the potential of different modes of delivery.

Despite these strengths, the review has limitations that must be acknowledged. One of the main limitations we faced was the difficulty in identifying BCTs from the included studies. Many studies did not clearly specify the BCTs they used, and in some cases, the behavioural intervention techniques were either vaguely described or not labelled according to the BCTTv1 taxonomy. Additionally, distinguishing between BCTs was difficult because some BCTs share similar descriptions, which required careful interpretation to ensure consistency in coding across the review team. An example is the two BCTs, ‘pros and cons’ and ‘information on health consequences’; both state the effects of the unwanted behaviour and promote the benefits of the desired behaviour. While we implemented strict guidelines to maintain consistency, this potential ambiguity remains a limitation in the accuracy of BCT identification. A previous review of smoking cessation interventions highlighted a limitation that the components used in the identified studies were not clearly stated (Moafa et al., 2021).

We also limited the review to studies available in English, which may have led to the exclusion of relevant studies published in other languages. This language bias could reduce the generalizability of the findings, particularly for interventions developed and tested in non-English-speaking countries. Additionally, we did not include qualitative studies or grey literature, which may have excluded valuable insights into the subjective experiences of participants or innovative intervention strategies that are not published in peer-reviewed journals.

The underrepresentation of certain BCTs within the reviewed studies also presents a limitation. Some BCTs (such as behavioural contract) were only used in one or two studies, limiting our ability to generalise their effectiveness. Given the increasing use of digital interventions, this is an area that needs further exploration in future studies. We must also consider these associations relate to outcomes but are not necessarily causative because of the nature of the included studies and the lack of published mediating and moderating analysis of these BCTs.

## Conclusion

Our review highlights the importance of several key BCTs, including goal setting, action planning, problem solving, and pharmacological support, in interventions that target both alcohol and tobacco use. Future research should continue to explore the optimal combinations of BCTs to maximise the effectiveness of interventions. While face-to-face interventions remain the most common mode of delivery, digital interventions hold considerable promise for the future, particularly as healthcare increasingly moves towards more technology-driven approaches. Future research should continue to refine and expand the use of these BCTs, with a focus on optimising delivery methods to enhance the overall effectiveness of interventions targeting the dual use of alcohol and tobacco.

## Author contributions

Conceptualization, N.M., S.V., and P.S.; Data curation, T.R., A.C, N.M., and K.M.; Formal analysis: A.C., K.M., D.V., M.P., and N.M.; Funding acquisition, N.M., S.V., and P.S.; Methodology, A.C., N.M., and S.V.; Resources, N.M., A.C., K.M., and T.R.; Writing – original draft, A.C., N.M., and K.M.; Writing – review & editing, A.C., N.M., K.M., S.V., L.Z., C.H., T.R., J.R., D.V., M.P., and P.S.

## Supplementary Material

Dual Use PRISMA_2020_checklist.docx

Appendix B

Appendix A

## Data Availability

Data from this study is available upon request.

## Open Scholarship

This article has earned the Center for Open Science badge for Preregistered. The materials are openly accessible at This study was registered with PROSPERO. (CRD42023445492).

## Systematic review registration

This study was registered with PROSPERO. (CRD42023445492)

## Analytic plan pre-registration

The analysis plan was registered in Prospero

## Analytic code

There is no analytic code associated with this study.

## Materials availability

Materials used to conduct this study are available here:

## BCT taxonomy tool

https://pubmed.ncbi.nlm.nih.gov/23512568/

## Covidence

https://www.covidence.org/

## Quality assessment tool

https://jbi.global/critical-appraisal-tools.
