# Supplementary material for: Behaviour change techniques reported in intervention studies of alcohol and tobacco use: a rapid review
Source: Health Psychol Behav Med. 2025 Sep 23;13(1):2554182. doi: 10.1080/21642850.2025.2554182 (PMC12459161; doi:10.1080/21642850.2025.2554182)
Supplement: Appendix B [file RHPB_A_2554182_SM8066.pdf]

Appendix B: Rapid Review Tables

Table 1. Summary of Identified Studies

Study (Author, Year)	Country of Study	Study Design, Sample Size, and Population	Intervention	Summary of BCTs	Outcome	Mode of Deliver y	Risk of Bias (Low, Moderate , or High)
Alba et al., 2022	Colombia	RCT<br>440 participants<br>*Individuals with upcoming surgical or diagnostic procedures	Intervention: Brief counseling intervention<br><br>Educational intervention	Goal Setting (Face to face/Printed material): Setting a reduction goal<br><br>Problem Solving (Face to face/Printed material): Identifying roadblocks for change<br><br>Monitoring of behaviour by others without feedback (Over the phone): Nurse called participants at 1 and 3-month intervals using standardized questionnaire in	Participants reported cessation of smoking and decreased alcohol consumption in both intervention groups	Face to face<br><br>Printed Material<br><br>Over the phone	Moderate

				assessing their consumption since intervention<br><br>Information about health consequences (Face to face/Printed material): Discussing effects of smoking and drinking, benefits of reducing smoking and drinking, and reading standard document about consequences<br><br>Information about social and environmental consequences (Face to face/Printed material): Discussing effects of smoking and			

				drinking, benefits of reducing smoking and drinking, and reading standard document about consequences			
Ames et al., 2010	USA	RCT<br>41 Participants<br>* Young adults	Control: Standard smoking treatment<br><br>Intervention: Standard smoking treatment combined binge-drinking intervention	Goal setting (Face to face): Setting personalized goals<br><br>Problem solving (Face to face): Identifying the role alcohol has played in smoking and may have affected prior quit attempts<br><br>Action planning (Face to face): Developing relapse prevention plan and strategies to overcome challenges with	A greater number of participants in the integrated intervention were biochemically confirmed abstinent from tobacco than the standard treatment and change from baseline in binge drinking episodes, drinks consumed and drinking days were similar among both groups	Face to face	Moderate

				maintain abstinence<br><br>Feedback on behaviour: Personalized feedback on consumption<br><br>Self-monitoring of behaviour: Self-monitoring of the relation of alcohol and tobacco use<br><br>Social comparison: Consumption compared with peer norms<br><br>Pharmacological support (Face to face): Nicotine patch therapy was used			
Ames et al., 2014	USA	RCT<br>95 Participants<br>*Young adults	Control: Individually based smoking cessation	Goal setting (Face to face): Setting personalized goals	Biochemically confirmed smoking abstinence rate at 6 months 21%	Face to face	Moderate

			Intervention: Individually based smoking cessation combined binge-drinking intervention	Problem solving (Face to face): Identifying the role alcohol has played in smoking and may have affected prior quit attempts<br><br>Action planning (Face to face): Developing relapse prevention plan and strategies to overcome challenges with maintain abstinence<br><br>Feedback on behaviour: Personalized feedback on consumption<br><br>Self-monitoring of behaviour: Self-monitoring of the relation of	(integrated intervention) and 9% (standard treatment) Average use of alcohol was lower at 6-months compared to baseline		

				alcohol and tobacco use<br><br>Feedback on outcome of behaviour (Face to face): Personalized feedback about use of alcohol<br><br>Social comparison: Feedback about consumption compared with peer norms<br><br>Pharmacological support (Face to face): Nicotine patch therapy was used			
Ayodapo & Olukokun, 2019	Nigeria	RCT<br>322 participants<br>*Adult hypertensive participants	Control: Lifestyle behaviour questionnaire<br><br>Intervention: Counselling on lifestyle behaviours	Goal setting (Face to face): Use of Five A's (Ask, Assess, Advise, Assist, Arrange) technique to moderate	100% of participants in the intervention group met the recommendation for alcohol consumption<br>84% of participants in	Face to face<br><br>Over the phone<br><br>SMS text	Moderate

				alcohol and smoking<br><br>Action Planning (Over the phone/ SMS text): Participants who did not meet the recommendation for alcohol or smoking were advised to write change plan<br><br>Social support (Over the phone/ SMS text): Ask help from their spouse for stopping their behaviour<br><br>Prompts/cues (Over the phone/ SMS text): Every two weeks reinforced of counselling through reminders over 12 weeks	the intervention group met the recommendation s for smoking		

				Behaviour substitution (Over the phone/ SMS text): Participants who did not meet the recommendation for alcohol or smoking were advised to obtain a substitute for alcohol/smoking<br><br>Distraction (Over the phone/ SMS text): Some participants were advised to spend their leisure time on religious activities			
Baumann et al., 2015	Denmark	RCT<br>9415 Participants<br>*General population	Control: Only-Assessment<br><br>Intervention: Individual life-style counselling	Goal setting (Face to face): Counselling sessions included goal setting strategies<br><br>Pros and cons: Counselling sessions targeted	Smoking abstinence remained significant 5 years after the intervention, average alcohol consumption did not change, but greater	Face to face	Moderate

				decisional balance the pros and cons for change<br><br>Social support (Face to face): Offered group-based counselling	reductions in binge drinking		
Burling et al., 2001	USA	RCT<br>200 Participants<br>*Drug- and alcohol-dependent cigarette smokers in a residential rehabilitation program for homeless veterans	Control:<br>Usual Care<br><br>Intervention:<br>Multicomponent smoking treatment plus generalization training of smoking cessation to drug and alcohol cessation	Goal setting: Employed a patient self-government and three-phase advancement system to monitor progress toward treatment goals<br><br>Problem solving (Face to face): Participants identified high risk situations relation to their consumption, learned and practiced coping skills<br><br>Action Planning:	Smoking abstinence rates were higher in the intervention group than the control at the 1-month post-quit point. Abstinence rates for alcohol decreased over time in all study groups.	Face to face	Moderate

				CB skills (assertion, conflict resolution, cognitive restructuring, active listening, relapse prevention) were taught<br><br>Behavioural contract (Face to face): One-to-one counselling sessions that included contingency contracting<br><br>Non-specific reward: Participants were granted additional privileges if contingent on demonstration of CB skill proficiency and progress to treatment goals			

				Pharmacological support (Face to face): Nicotine patches and/or gum were used<br><br>Negative reinforcement (Face to face): Random and weekly breath and urine tests were conducted if any were present discharged from the domiciliary			
Burton et al., 1995	USA	RCT<br>3097 Participants<br>*Elderly population with Medical Insurance and a primary care physician participating in the study	Control:<br>Usual care<br><br>Intervention:<br>Preventive and counselling visits	Encourage older patients to improve health-related behaviors, including smoking cessation, reduction of problem drinking<br><br>During preventive visit review and/or discussion of	Higher proportion of participants quit smoking in the intervention compared to the control, higher proportion of problem drinkers in the control group improved	Face to face	High

				smoking status review and/or discussion of problem alcohol drinking			
Correa-Fernández et al., 2017	Puerto Rico	RCT<br>202 Participants<br>* Smokers who were attempting to quit smoking and who were also at-risk drinkers	Control: Standard Motivation and Problem Solving (MAPS)<br><br>Intervention: MAPS+, focused on cessation and at-risk drinking reduction	Goal setting (Over the phone): Setting a quit date<br><br>Problem solving (Over the phone): Used problem solving techniques to help participants in maintaining their quit attempts<br><br>Action planning (Over the phone): Used skills training in initiating, maintaining quit attempts and recovering from relapses<br><br>Review behaviour goal (Over the	No changes in smoking abstinence. Intervention group increased the level of reducing at-risk drinking behaviours compared to control group (only among smokers who successfully quit)	Over the phone<br><br>Printed Material	Moderate

				phone): Helps participants guide through the course of the treatment to alternate motivational to skill-building focus<br><br>Information about Antecedents (Over the phone): If topic of alcohol didn’t surface, the counselor will state alcohol is a common antecedent of smoking and inquire about it			
Duffy et al., 2006	USA	RCT<br>184 Participants<br>*Head and Neck cancer patients	Control:<br>Usual care<br><br>Intervention:<br>Nurse Administered intervention	Goal setting (Over the phone): The CBT approaches emphasises goal setting<br><br>Problem solving (Over the phone): The	47% of participants in the intervention group quit smoking and 1/3 of problem drinking was reduced in all groups.	Over the phone<br><br>Face to face<br><br>Printed Material	Moderate

				CBT approaches emphasizes coping skills<br><br>Action Planning (Over the phone): The CBT approaches emphasizes coping skills and social skills training<br><br>Self-monitoring of behaviour: The CBT approaches emphasizes self-monitoring<br><br>Information about Antecedents (Over the phone): The CBT approaches emphasizes analyzing behavioural antecedents<br><br>Information about health			

				consequences (Over the phone): Workbook included concepts covering the association of head and neck cancer with smoking, depression and alcohol<br><br>Pharmacological support (Face to face): Offered NRT and/or bupropion			
Frank et al., 2007	USA	Case-Control<br>224 Participants<br>*Medical students	Control:<br>No intervention administrated to Class of 2002<br><br>Intervention:<br>4-year intervention to promote healthy behaviors among students in Class of 2003	Information about health consequences: Lectures on pathophysiology of hepatic diseases emphasizing students personal consumption practices	Control group males tobacco use increased while intervention group decreased. No difference in alcohol consumption	Face to face<br><br>Printed Material	Low

				Credible source: Additional lectures on nondidactic exposures about various topics including tobacco, alcohol presented by national leaders In these fields			
Fucito et al., 2020	USA	RCT<br>26 Participants<br>*General population who smoke and drink alcohol and were interested in changing either behaviour.	Intervention: Integrated tobacco and alcohol counselling	Goal setting: Counselling sessions incorporated evidence based content based on alcohol and tobacco included goal setting<br><br>Feedback on behavior: At three time points (intake, midpoint, termination) participants received feedback on their tobacco and/or alcohol	Double the participants in the intervention group quit smoking compared to control and percentage of heavy drinking days decreased in all groups	Face to face	Moderate

				use and health status<br><br>Information about antecedents: Managing urges and triggers<br><br>Pharmacological support: Varenicline (2mg) for 12 weeks			
Ganavadiya et al., 2018	India	RCT<br>83 Participants<br>*Patients with addiction to tobacco and alcohol	Intervention: Motivation interview<br><br>Games & Story therapy<br><br>Reading & Writing therapy	Social support: Group counselling sessions<br><br>Information about antecedents: Reasons for addiction were discussed in group counseling sessions<br><br>Information about health consequences (Face to face):	Mean Fagerstrom Test for Nicotine Dependence (FTND) scores decreased in all intervention groups compared to baseline<br><br>Could not assess alcohol dependence	Face to face<br><br>Printed Material	Moderate

				Adverse medical and social consequences attributable to addiction were discussed<br><br>Demonstration of the behaviour: Use of story therapy to demonstrate addiction<br><br>Social comparison: An anonymous meeting from a discharged person was held to share their experience<br><br>Comparative imagining of future outcomes (Face to face): Lifestyle post-discharge were discussed<br><br>Distraction (Face to face):			

				Games therapy was used to divert participants that may mask craving and withdrawal symptoms<br><br>Verbal persuasion about capability: Use of anonymous meetings to facilitate change in attitude and behaviour			
Gray et al., 2005	United Kingdom	Case-Control<br>140 Participants<br>*Students	Control: Assessment only<br><br>Intervention: Motivational interview	Motivational Interviewing MI principles: Use of reflective listening, asking open-ended questions, providing summaries and affirmations	No significant reduction in smoking consumption<br><br>Alcohol consumption decreased in intervention group compared to control group	Face to face<br><br>Over the phone<br><br>Mail	High
Hawkes et al., 2013	Australia	RCT<br>410 Participants<br>*Cancer Patients	Control: Usual care<br><br>Intervention:	Goal setting (Over the phone): Sessions included goal setting	No differences in smoking and alcohol outcomes	Over the phone<br><br>Printed Material	Moderate

			Health coaching intervention	Problem solving (Over the phone): Sessions included problem solving<br><br>Action Planning (Over the phone): Sessions included action planning<br><br>Monitoring of behaviour by others without feedback (Over the phone): Sessions included reviewing and ongoing monitoring of health behaviours<br><br>Information about health consequences: Received participant handbook and educational			

				brochures produced by Cancer Council Australia			
Joseph et al., 2003	USA	RCT<br>499 Participants<br>*Current smokers in intensive alcohol dependence treatment	Intervention: Smoking cessation concurrently with alcohol treatment<br>or six months after treatment	Goal setting: Asked participants to set a quit date<br><br>Problem solving: Evaluated barriers to quitting<br><br>Social support: Provided support for change<br><br>Pros and cons: Provided information about the pros and cons of smoking (including interaction between smoking and alcohol)<br><br>Pharmacological support (Face to	No differences in smoking and alcohol outcomes	Face to face<br><br>Over the phone	Moderate

				face): Prescribed NRT			
Joseph et al., 2004	USA	RCT<br>499 Participants<br>*Current smokers in intensive alcohol dependence treatment	Intervention: Concurrent smoking intervention or delayed	Addressed the relationships between smoking and alcohol use and the potential advantages of recovery from all substance use<br><br>Pharmacological support (Face to face): Prescription of NRT	45% of participants claimed to be abstinent from smoking<br><br>Alcohol abstinence was lower in the concurrent treatment group than the delayed treatment group	Face to face<br><br>Over the phone	Low
Kahler et al., 2008	USA	RCT<br>236 Participants<br>*Heavy drinking smokers recruited from the community.	Control: Standard smoking cessation treatment<br><br>Intervention: Incorporated brief alcohol intervention	Goal setting: Encouraged to abstain from alcohol 2 weeks from quitting smoking while on nicotine patch<br><br>Feedback on behaviour: Normative feedback on drinking level and the risk of smoking relapse	No difference in smoking abstinence rates among both groups<br><br>Great reduction in weekly alcohol consumption in both conditions	Face to face	Low

				associated with drinking<br><br>Information about health consequences: Informed of the risks associated with combined heavy drinking and smoking<br><br>Pharmacological support: All participants received transdermal nicotine patch			
Koelewijn-van Loon et al., 2009	Netherlands	RCT<br>615 Participants<br>*Adult patients eligible for cardiovascular risk management	Control: Minimal nurse-led intervention<br><br>Intervention: nurse-led cardiovascular risk management	Goal setting (Face to face): Used adapted motivational interviewing to achieve goal setting<br><br>Action Planning: Used adapted motivational interviewing for concrete action plans	No differences in smoking and alcohol outcomes	Face to face	Moderate

				Pros and cons: Used adapted motivational interviewing to reveal both positive and negative consequences of behaviour change			
Lauridsen et al., 2022	Denmark	RCT<br>94 Participants<br>*Patients scheduled for radical cystectomy for bladder cancer and who smoked daily and/or consumed at least 3 units of alcohol (36 g) daily	Control:<br>Usual care<br><br>Intervention:<br>Intensive smoking and/or alcohol cessation intervention	Pharmacological support: Offered NRT, chlordiazepoxide and low-dose disulfiram<br><br>Pros and cons: During first meeting before admission and fourth meeting benefits of long-term smoking and/or alcohol abstinence were discussed<br><br>Information about health consequences: Dependence	Number of participants in the intervention and control group were abstinent from smoking and reduced their alcohol intake	Face to face<br><br>Printed Material	Moderate

				withdrawal symptoms were discussed			
Lim et al., 2018	USA	RCT<br>22 Participants<br>*Treatment-seeking smokers	Control: smoking cessation counseling only<br><br>Intervention: Brief smoking cessation intervention addressing alcohol use	Goal setting: Set a smoking quit date within the 30 days post intervention<br><br>Problem solving: Participants were asked to identify recurrent situations in which they experienced craving<br><br>Action planning: Work together to develop methods for coping with cravings<br><br>Feedback on behaviour: Received personalized feedback on their cigarette use and drinking patterns	3 participants out of 11 in each intervention met smoking abstinence at 1 month follow up and 1 participant in each intervention indicated stable reduction in alcohol use	Face to face	Moderate

				Behavioural practice rehearsal: Provided brief psychoeducation on the selected skills and encouraged to discuss and practice the skills<br><br>Pros and cons: Discussed pros and cons of smoking and quitting and reducing alcohol use while quitting smoking			
Louwagie et al., 2022	South Africa	RCT<br>574 Participants<br>*Adults starting treatment for drug-<br>sensitive pulmonary TB who smoked tobacco or reported harmful/hazardou s alcohol use	Intervention: Brief motivational interviewing sessions	Goal setting (Face to face): Involved prioritisation and agenda setting<br><br>Action planning: Determined which factor should be prioritised	Participants in the intervention and control abstained from smoking<br><br>AUDIT scores were lower in follow-up compared to baseline	Face to face<br><br>SMS text	Moderate

Macleod et al., 2018	United Kingdom	Case-control<br>22 Participants<br>*Cancer Patients	Intervention: Counselling sessions	Goal setting: Personalized specific action goals were identified with a focus on two health behaviours prioritized by the individual<br><br>Action Planning: Participants were encouraged to develop personalized action and coping plans<br><br>Self-monitoring of behaviour: Emphasis was placed on self-monitoring<br><br>Social support: Participants were encouraged to invite a support person to help with the program	No change in smoking outcome, alcohol consumption decreased	Face to face<br><br>Over the phone<br><br>Printed Material	High

				Demonstration of the behaviour: Activities such as brisk walking were demonstrated and attempted by participants			
Minian et al., 2019	Canada	RCT<br>5715 Participants<br>*<br>Patients in a smoking cessation program. reporting consuming alcohol above guidelines	Control:<br>No computer alerts<br><br>Intervention:<br>Received prompt when a patient reported consuming alcohol above the guidelines	Prompts/cues: Practitioners were prompted to provide brief alcohol reduction or abstinence intervention<br><br>Pharmacological support: Use of NRT	112 participants out of 1332 in intervention and 121 out of 1346 in control abstained from smoking and drinking	Web	Low
McCambridge et al., 2011	United Kingdom	RCT<br>416 Participants<br>*Students	Control:<br>Standard practice<br><br>Intervention:<br>Individualized motivational interviewing	Problem solving: Encouraged to think and discuss a series of hypothetical situations to refuse offers of drugs<br><br>Action planning: Explored the reasons for not	No differences in smoking and alcohol outcomes	Face to face	Moderate

				using specific substances and how initiation of use might affect future plans			
McCambridge et al., 2005	United Kingdom	RCT<br>200 Participants<br>*Young people who were current users of illegal drugs	Control: Assessment only<br><br>Intervention: Motivational interviewing	Discussion about the relationships with drugs<br><br>Participants choose which drugs were worthy to focus using the objects of the intervention	Mean smoking consumption decreased in intervention group<br>Frequency of alcohol consumption decreased in intervention group at 3 months	Face to face	Moderate
McCambridge et al., 2003	United Kingdom	RCT<br>179 Participants<br>*Young people who were current users of illegal drugs	Control: Usual care<br><br>Intervention: Motivational interviewing	Goal setting (Face to face): Explored non-drug values and goals<br><br>Problem solving (Face to face): Identification of problems and concerns<br><br>Information about health consequences (Face to face):	No differences in smoking and alcohol outcomes	Face to face	Moderate

				Focused the areas of risk problems or concerns			
Meacham et al., 2021	USA	RCT<br>179 Participants<br>*Young adults	Control:<br>Tobacco-only intervention<br><br>Intervention:<br>Smoking Tobacco and Drinking intervention	Problem solving (Web): Posts included coping with cravings, refusal skills, planning for emergencies and managing negative moods<br>Action planning (Web): Posts included coping with cravings, refusal skills, planning for emergencies and managing negative moods<br><br>Feedback on behaviour (Web): Provided normative feedback on consumption patterns<br><br>Information about	Abstinence from smoking in all groups showed no significant difference and no significant difference in heavy drinking episodes in all groups	Web<br><br>Mail	Moderate

				antecedents (Web): Posts elicited thoughts about how alcohol use may have affected smoking behaviours and quit attempts<br><br>Prompts/cues (Web): Tailored prompts and content for readiness to quit<br><br>Pharmacological support (Mail): Offered starter packs of nicotine patches			
Nguyen et al., 2012	Vietnam	Case-control<br>4650 Participants<br>*General population	Control: Conventional healthcare services<br><br>Intervention: Hypertensive targeted management programme	Mass media campaigns: Lifestyle promotion campaigns through broadcasting focused on smoking cessation and reducing alcohol consumption	No changes in smoking outcomes and reduction in heavy alcohol consumption in all study conditions	Mass media	High

Pengpid et al., 2015	Thailand	RCT<br>620 Participants<br>*Patients who met the cut off for moderate risk of conjoint alcohol and tobacco use	Control:<br>Alcohol only or tobacco only intervention<br><br>Intervention:<br>Co-joint intervention	Feedback on behaviour:<br>Feedback and discussion on the relationship between alcohol and smoking<br><br>Information about antecedents:<br>Discussed potential effects of alcohol consumption on smoking cessation	Consistent improvements in all outcome measures among study groups	Face to face	Moderate
Ravindranath et al., 2018	India	RCT<br>1208 Participants<br>*General population	Intervention:<br>Sessions targeting cardiometabolic risk factors	Goal setting:<br>Education sessions included participant workbook on goal setting<br><br>Self-monitoring of behaviour:<br>Education sessions included participant workbook to guide self-	Tobacco and alcohol consumption decreased	Face to face	Moderate

				monitoring of lifestyle behaviours<br><br>Social support: Education sessions were conducted via peer groups and were encouraged to participate in health promotion activities (kitchen gardening, yoga sessions and walking groups)			
Sabari Sridhar et al., 2017	India	Case-control<br>200 Participants<br>*Patients aged between 18 and 75 years, who use tobacco/alcohol	Control: Questionnaire and information pamphlet<br><br>Intervention: Brief intervention for both tobacco and alcohol cessation	Feedback on behaviour: Report card given for personalized feedback and associated health problems<br><br>Information about health consequences (Face to face): Discussed associated health problems	Statistically significant reduction in tobacco and alcohol from baseline to follow-up in both groups	Face to face	Low

Sobell et al., 2017	USA	RCT<br>317 Participants<br>*Military primary care center patients	Control: Standard smoking cessation<br><br>Intervention: Smoking cessation with alcohol reduction	Goal setting (Face to face): Established quit date<br><br>Problem solving (Over the phone/ Workbook): Identified triggers and went over problem solving techniques to overcome difficulties<br><br>Action planning (Face to face): Given framework to analyzing their consumption and developing a change plan<br><br>Feedback on behaviour (Face to face): Received feedback about their consumption	Higher smoking cessation rates in intervention group compared to control group and reduced alcohol consumption in both groups	Face to face<br><br>Over the phone<br><br>Printed Material	Moderate

				with national norms<br><br>Information about health consequences (Face to face): Provided risks about excessive drinking and calories consumed<br><br>Pros and cons (Face to face): Discussed barriers and benefits of change<br><br>Pharmacological support: Provided NRT and/or Zyban			
Stotts et al., 2003	USA	RCT<br>115 Participants<br>*Patients with consecutive admissions to the Treatment Research Clinic (TRC) in Houston, TX	Intervention: Dual-substance dependence program	Pharmacological support:<br>Alcohol (Naltrexone or Placebo)<br>Smoking (Transdermal NRT or Placebo)	No differences in smoking and alcohol outcomes	Face to face	High

Toll et al., 2015	USA	RCT<br>1948 Participants<br>*Heavy-drinking adult smokers	Control:<br>Standard care<br><br>Intervention:<br>Alcohol and tobacco counselling	Goal setting (Over the phone): Asked to set a goal and based off research requested to reduce alcohol consumption<br><br>Information about health consequences (Over the phone): Discussed effects of drinking and smoking<br><br>Information about antecedents (Over the phone): Discussed how alcohol might affect effort to quit smoking<br><br>Pharmacological support (Mail): Offered starter	Intervention group had higher rate of smoking abstinence than control and reported fewer heavy drinking days than control	Over the phone<br><br>Mail	Moderate

				package of nicotine patches or gum			
Vander Weg et al., 2016	USA	RCT<br>63 Participants<br>*Rural Veteran daily cigarette smokers who were interested in quitting and received care at a Midwestern VA Medical Center	Control: Referral to Quitline<br><br>Intervention: Tailored telephone counselling	Goal setting (Over the phone): Discussed preparedness to quit and allowed to set their own goals<br><br>Problem solving (Over the phone): Discussed quitting process, maintain short-term abstinence and relapse prevention<br><br>Action planning (Over the phone): Discussed quitting process, maintain short-term abstinence and relapse prevention	Smoking quit rates higher in the intervention group than in control, but no statistically significant differences in group and Participants who were eligible to participate in the alcohol risk reduction intervention did not agree to participate	Over the phone	Low

				Pharmacological support: Offered several forms of NRT, bupropion and varenicline			
Vinci et al., 2023	USA	RCT<br>67 Participants<br>* Adults who smoked cigarettes who reported binge drinking and were motivated to both quit smoking and change their alcohol use.	Intervention: Mindfulness-Based Relapse Prevention for Smoking and Alcohol<br><br>Cognitive Behavioral Therapy	Goal setting (App): Preparing participants for quitting smoking and implementing their alcohol change goal<br><br>Action planning (App): Used traditional CBT skills for identifying triggers , coping skill development, cognitive restructuring<br><br>Social support (Web): Weekly group sessions<br><br>Pharmacological support: Received	Tobacco use and heavy drinking days were reduced in each group significantly	App<br><br>Web<br><br>Printed Material	Moderate

				NicoDerm CQ patch			
Vrdoljak et al., 2014	Croatia	RCT<br>738 Participants<br>*Citizens of Croatia aged 65 years who visited their GP for any reason	Control:<br>Usual care<br><br>Intervention:<br>Intensified intervention	Problem solving (educational leaflets):<br>Received instructions on using non-pharmacological and/or pharmacological intervention and in accordance to the guidelines when risk factors are detected<br><br>Action planning:<br>If cardiovascular risk factors were detected, participants were counselled an individual plan for adopting healthier life habits<br><br>Instruction on how to perform the behaviour:<br>Additional	Few smokers stopped smoking, and no changes in drinking consumption	Face to face<br><br>Printed Material	Low

				training on using non-pharmacological and/or pharmacological interventions were provided			
Witkiewitz et al., 2014	USA	RCT<br>94 Participants<br>*Students	Control: Mobile assessments only<br>Intervention: Mobile intervention	Action planning (Over the phone): Suggestions for less risky drinking habits and brainstorming alternatives to heavy drinking<br><br>Feedback on behaviour (Over the phone): Personalized and normative feedback about consumption behaviour<br><br>Information about health consequences (Over the phone): Educated the	No differences in smoking and alcohol outcomes	Web	Moderate

				effects of alcohol on the brain and behaviour<br><br>Behaviour substitution (Over the phone): Discussed alternative activities to drinking and smoking			

Table 2. BCTs Activity Frequency of Low & Moderate Bias Studies

BCT Activities<br>*Mode of Delivery	Number of Studies Used	Only Tobacco Reduction	Only Alcohol Reduction	Reduction for Both
**Goal Setting**	21	3	2	12
*Face to Face	16	2	1	10
*Digital (Over the Phone, SMS, App, Web)	11	1	1	8
*Printed Material	6	0	1	4
*Mail	1	0	0	1
**Action Planning**	17	3	1	8
*Face to Face	10	2	0	7
*Digital (Over the Phone, SMS, App, Web)	10	1	0	6
*Printed Material	6	2	1	3
**Pharmacological support**	15	2	1	10
*Face to Face	10	1	1	8
*Digital (Over the Phone, SMS, App, Web)	7	1	0	5
*Printed Material	4	0	0	4
*Mail	1	0	0	1
**Problem-Solving**	15	3	1	6
*Face to Face	11	2	0	6
*Digital (Over the Phone, SMS, App, Web)	6	1	1	4
*Printed Material	5	1	1	3
*Mail	1	0	0	1
**Information about health consequences**	12	2	1	6
*Face to Face	9	1	0	5
*Digital (Over the Phone, SMS, App, Web)	6	0	0	4
*Printed Material	7	2	0	4
*Mail	1	0	0	1
**Feedback on behaviour**	10	1	1	6

*Face to Face	8	1	1	6
*Digital (Over the Phone, SMS, App, Web)	3	0	0	1
*Printed Material	1	0	0	1
*Mail	1	0	0	0
**Information about antecedents**	7	1	1	4
*Face to Face	4	1	0	3
*Digital (Over the Phone, SMS, App, Web)	4	0	1	2
*Printed Material	3	1	1	1
*Mail	2	0	0	1
**Pros and cons**	6	1	0	3
*Face to Face	6	1	0	3
*Digital (Over the Phone, SMS, App, Web)	1	0	0	1
*Printed Material	2	0	0	2
**Self-monitoring of behaviour**	4	1	0	3
*Face to Face	4	1	0	3
*Digital (Over the Phone, SMS, App, Web)	1	0	0	1
*Printed Material	1	0	0	1
**Social support**	6	2	0	4
*Face to Face	5	2	0	2
*Digital (Over the Phone, SMS, App, Web)	2	0	0	2
*Printed Material	2	1	0	1
**Prompts/cues**	3	0	0	2
*Face to Face	1	0	0	1
*Digital (Over the Phone, SMS, App, Web)	4	0	0	2
*Mail	1	0	0	0
**Social comparison**	3	2	0	1
*Face to Face	3	2	0	1
*Printed Material	1	1	0	0

**Monitoring of behaviour by others without feedback**	2	0	0	1
*Face to Face	1	0	0	1
*Digital (Over the Phone, SMS, App, Web)	2	0	0	1
*Printed Material	2	0	0	1
**Behaviour substitution**	2	0	0	1
*Face to Face	1	0	0	1
*Digital (Over the Phone, SMS, App, Web)	3	0	0	2
**Distraction**	2	1	0	1
*Face to Face	2	2	0	1
*Digital (Over the Phone, SMS, App, Web)	2	0	0	2
*Printed Material	1	0	0	0
**Feedback on outcome of behavior**	1	0	0	1
*Face to Face	1	0	0	1
**Demonstration of the behaviour**	1	1	0	0
*Face to Face	1	1	0	0
*Printed Material	1	1	0	0
**Review behaviour goal**	1	0	1	0
*Digital (Over the Phone, SMS, App, Web)	1	0	1	0
*Printed Material	1	0	1	0
**Behavioral contract**	1	0	0	1
*Face to Face	1	0	0	1
**Instruction on how to perform the behaviour**	1	1	0	0
*Face to Face	1	1	0	0
*Printed Material	1	1	0	0
**Information about social and environmental consequences**	1	0	0	1
*Face to Face	1	0	0	1

*Digital (Over the Phone, SMS, App, Web)	1	0	0	1
*Printed Material	1	0	0	1
**Behavioural practice rehearsal**	1	0	0	1
*Face to Face	1	0	0	1
**Credible source**	1	1	0	0
*Face to Face	1	1	0	0
*Printed Material	1	1	0	0
**Comparative imagining of future outcomes**	1	1	0	0
*Face to Face	1	1	0	0
*Printed Material	1	1	0	0
**Non-specific reward**	1	0	0	1
*Face to Face	1	0	0	1
**Verbal persuasion about capability**	1	1	0	0
*Face to Face	1	1	0	0
*Printed Material	1	1	0	0
**Negative reinforcement**	1	0	0	1
*Face to Face	1	0	0	1

Table B3. BCT Activity of Statistically Significant Studies

BCT Activities<br>*Mode of Delivery	# of studies demonstrating only improved smoking cessation (N= 2)	# of studies demonstrating only alcohol reduction (N= 1)	# of studies demonstrating improved smoking cessation and alcohol reduction (N= 13)
**Goal Setting**	2<br>(100%)	1<br>(100%)	9<br>(69%)
*Face to Face	2	0	7
*Digital (Over the Phone, SMS, App, Web)	0	1	6
*Printed Material	0	1	3
**Action Planning**	1<br>(50%)	1<br>(100%)	6<br>(46%)
*Face to Face	1	0	5
*Digital (Over the Phone, SMS, App, Web)	0	1	4
*Printed Material	0	1	2
**Pharmacological support**	1<br>(50%)	0	8<br>(62%)
*Face to Face	1	0	6
*Digital (Over the Phone, SMS, App, Web)	0	0	4
*Printed Material	0	0	3
**Problem-Solving**	1<br>(50%)	1<br>(100%)	5<br>(38%)
*Face to Face	1	0	5
*Digital (Over the Phone, SMS, App, Web)	0	1	3
*Printed Material	0	0	2
**Information about health consequences**	0	0	6<br>(46%)

*Face to Face	0	0	5
*Digital (Over the Phone, SMS, App, Web)	0	0	4
*Printed Material	0	0	3
**Feedback on behaviour**	1<br>(50%)	0	5<br>(38%)
*Face to Face	1	0	5
*Digital (Over the Phone, SMS, App, Web)	0	0	1
*Printed Material	0	0	1
**Information about antecedents**	0	1<br>(100%)	4<br>(31%)
*Face to Face	0	0	3
*Digital (Over the Phone, SMS, App, Web)	0	1	2
*Printed Material	0	1	0
**Pros and cons**	1<br>(50%)	0	2<br>(15%)
*Face to Face	1	0	2
*Digital (Over the Phone, SMS, App, Web)	0	0	1
*Printed Material	0	0	2
**Self-monitoring of behaviour**	1<br>(50%)	0	2<br>(15%)
*Face to Face	1	0	2
*Digital (Over the Phone, SMS, App, Web)	0	0	1
**Social support**	1<br>(50%)	0	2<br>(15%)
*Face to Face	1	0	1

*Digital (Over the Phone, SMS, App, Web)	0	0	2
*Printed Material	0	0	1
**Prompts/cues**	0	0	1<br>(8%)
*Face to Face	0	0	1
*Digital (Over the Phone, SMS, App, Web)	0	0	1
**Social comparison**	1<br>(50%)	0	1<br>(8%)
*Face to Face	1	0	1
**Monitoring of behaviour by others without feedback**	0	0	1<br>(8%)
*Face to Face	0	0	1
*Digital (Over the Phone, SMS, App, Web)	0	0	1
*Printed Material	0	0	1
**Behaviour substitution**	0	0	1<br>(8%)
*Face to Face	0	0	1
*Digital (Over the Phone, SMS, App, Web)	0	0	1
**Distraction**	0	0	1<br>(8%)
*Face to Face	0	0	1
*Digital (Over the Phone, SMS, App, Web)	0	0	1
**Feedback on outcome of behavior**	0	0	1<br>(8%)
*Face to Face	0	0	1

**Review behaviour goal**	0	1<br>(100%)	0
*Digital (Over the Phone, SMS, App, Web)	0	1	0
*Printed Material	0	1	0
**Behavioral contract**	0	0	1<br>(8%)
*Face to Face	0	0	1
**Information about social and environmental consequences**	0	0	1<br>(8%)
*Face to Face	0	0	1
*Digital (Over the Phone, SMS, App, Web)	0	0	1
*Printed Material	0	0	1
**Non-specific reward**	0	0	1<br>(8%)
*Face to Face	0	0	1
**Negative reinforcements**	0	0	1<br>(8%)
*Face to Face	0	0	1

Table 4. Summary of Outcome and Mode of Delivery

Study	BCTs	Tobacco Outcome	Alcohol Outcome	Mode of Delivery
Alba et al., 2022	Goal Setting<br><br>Problem Solving<br><br>Monitoring of behaviour by others without feedback<br><br>Information about health consequences<br><br>Information about social and environmental consequences	Cessation of smoking in both intervention groups<br><br>Reduction	Low alcohol consumption in both intervention groups<br><br>Reduction	Face to face<br><br>Printed Material<br><br>Over the phone
Ames et al., 2010	Goal setting<br><br>Problem solving<br><br>Action planning<br><br>Feedback on behaviour<br><br>Self-monitoring of behaviour<br><br>Social comparison<br><br>Pharmacological support	Biochemically confirmed abstinent from tobacco<br><br>Reduction	Baseline in binge drinking episodes, drinks consumed and drinking days were similar among both groups<br><br>No Reduction	Face to face

**Study**	**BCTs**	**Tobacco Outcome**	**Alcohol Outcome**	**Mode of Delivery**
Ames et al., 2014	Goal setting<br><br>Problem solving<br><br>Action planning<br><br>Feedback on behaviour<br><br>Self-monitoring of behaviour<br><br>Feedback on outcome of behaviour<br><br>Social comparison<br><br>Pharmacological support	Biochemically confirmed smoking abstinence rate at 6 months 21% (integrated intervention) and 9% (standard treatment)<br><br>Reduction	Average use of alcohol was lower at 6-months compared to baseline<br><br>Reduction	Face to face
Ayodapo & Olukokun, 2019	Goal setting<br><br>Action Planning<br><br>Social support<br><br>Prompts/cues<br><br>Behaviour substitution<br><br>Distraction	84% of participants in the intervention group met the recommendations for smoking<br><br>Reduction	100% of participants in the intervention group met the recommendation for alcohol consumption<br><br>Reduction	Face to face<br><br>Over the phone<br><br>SMS text

**Study**	**BCTs**	**Tobacco Outcome**	**Alcohol Outcome**	**Mode of Delivery**
Baumann et al., 2015	Goal setting<br><br>Pros and cons<br><br>Social support	Smoking abstinence remained significant 5 years after the intervention<br><br>Reduction	Average alcohol consumption did not change, but greater reductions in binge drinking<br><br>No reduction	Face to face
Burling et al., 2001	Goal setting<br><br>Problem solving<br>Action Planning<br><br>Behavioural contract<br><br>Non-specific reward<br><br>Pharmacological support<br><br>Negative reinforcement	Smoking abstinence rates were higher in the intervention group than the control at the 1-month post-quit point.<br><br>Reduction	Abstinence rates for alcohol decreased over time in all study groups.<br><br>Reduction	Face to face
Correa-Fernández et al., 2017	Goal setting<br><br>Problem solving<br><br>Action planning<br><br>Review behaviour goal<br><br>Information about Antecedents	No changes in smoking abstinence<br><br>No Reduction	Intervention group increased the level of reducing at-risk drinking behaviours compared to control group<br><br>Reduction	Over the phone<br><br>Printed Material

Study	BCTs	Tobacco Outcome	Alcohol Outcome	Mode of Delivery
Duffy et al., 2006	Goal setting<br><br>Problem solving<br><br>Action Planning<br><br>Self-monitoring of behaviour<br><br>Information about Antecedents<br><br>Information about health consequences<br><br>Pharmacological support	47% of participants in the intervention group quit smoking<br><br>Reduction	1/3 of problem drinking was reduced in all groups.<br><br>Reduction	Over the phone<br><br>Face to face<br><br>Printed Material
Frank et al., 2007	Information about health consequences<br><br>Credible source	Control group males tobacco use increased while intervention group decreased.<br><br>Reduction	No difference in alcohol consumption<br><br>No reduction	Face to face<br><br>Printed Material
Fucito et al., 2020	Goal setting<br><br>Feedback on behavior<br><br>Information about antecedents<br><br>Pharmacological support	Double the participants in the intervention group quit smoking compared to control<br><br>Reduction	Percentage of heavy drinking days decreased in all groups<br><br>Reduction	Face to face

Study	BCTs	Tobacco Outcome	Alcohol Outcome	Mode of Delivery
Ganavadiya et al., 2018	Social support<br><br>Information about antecedents<br><br>Information about health consequences<br><br>Demonstration of the behaviour<br><br>Social comparison<br><br>Comparative imagining of future outcomes<br><br>Distraction<br><br>Verbal persuasion about capability	Mean Fagerstrom Test for Nicotine Dependence (FTND) scores decreased in all intervention groups compared to baseline<br><br>Reduction	Could not assess alcohol dependence<br><br>No reduction	Face to face<br><br>Printed Material
Hawkes et al., 2013	Goal setting<br><br>Problem solving<br><br>Action Planning<br>Monitoring of behaviour by others without feedback	No differences in smoking and alcohol outcomes<br><br>No reduction	No differences in smoking and alcohol outcomes<br><br>No reduction	Over the phone<br><br>Printed Material

Study	BCTs	Tobacco Outcome	Alcohol Outcome	Mode of Delivery
	Information about health consequences			
Joseph et al., 2003	Goal setting<br><br>Problem solving<br><br>Social support<br><br>Pros and cons<br><br>Pharmacological support	No differences in smoking and alcohol outcomes<br><br>No reduction	No differences in smoking and alcohol outcomes<br><br>No reduction	Face to face<br><br>Over the phone
Joseph et al., 2004	Addressed the relationships between smoking and alcohol use and the potential advantages of recovery from all substance use<br><br>Pharmacological support	45% of participants claimed to be abstinent from smoking<br><br>Reduction	Alcohol abstinence was lower in the concurrent treatment group than the delayed treatment group<br><br>Reduction	Face to face<br><br>Over the phone
Kahler et al., 2008	Goal setting<br><br>Feedback on behaviour<br><br>Information about health consequences<br><br>Pharmacological support	No difference in smoking abstinence rates among both groups<br><br>No reduction	Great reduction in weekly alcohol consumption in both conditions<br><br>Reduction	Face to face

Study	BCTs	Tobacco Outcome	Alcohol Outcome	Mode of Delivery
Koelewijn-van Loon et al., 2009	Goal setting<br><br>Action Planning<br>Pros and cons	No differences in smoking and alcohol outcomes<br>No reduction	No differences in smoking and alcohol outcomes<br><br>No reduction	Face to face
Lauridsen et al., 2022	Pharmacological support<br><br>Pros and cons<br><br>Information about health consequences	Number of participants in the intervention and control group were abstinent from smoking<br><br>Reduction	Number of participants in the intervention and control group reduced their alcohol intake<br><br>Reduction	Face to face<br><br>Printed Material
Lim et al., 2018	Goal setting<br><br>Problem solving<br><br>Action planning<br><br>Feedback on behaviour	3 participants out of 11 in each intervention met smoking abstinence at 1 month<br><br>Reduction	1 participant in each intervention indicated stable reduction in alcohol use<br><br>Reduction	Face to face

Study	BCTs	Tobacco Outcome	Alcohol Outcome	Mode of Delivery
	Behavioural practice rehearsal<br><br>Pros and cons			
Louwagie et al., 2022	Goal setting<br><br>Action planning	Participants in the intervention and control abstained from smoking<br><br>Reduction	AUDIT scores were lower in follow-up compared to baseline<br><br>Reduction	Face to face<br><br>SMS text
Minian et al., 2019	Prompts/cues<br><br>Pharmacological support	112 participants out of 1332 in intervention and 121 out of 1346 in control abstained from smoking and drinking<br><br>Reduction	112 participants out of 1332 in intervention and 121 out of 1346 in control abstained from smoking and drinking<br><br>Reduction	Web
McCambridge et al., 2011	Problem solving<br><br>Action planning	No differences in smoking and alcohol outcomes<br><br>No reduction	No differences in smoking and alcohol outcomes<br><br>No reduction	Face to face
McCambridge et al., 2005	Discussion about the relationships with drugs<br><br>Participants choose which drugs were worthy to focus using the objects of the intervention	Mean smoking consumption decreased in intervention group<br><br>Reduction	Frequency of alcohol consumption decreased in intervention group at 3 months<br><br>Reduction	Face to face

Study	BCTs	Tobacco Outcome	Alcohol Outcome	Mode of Delivery
McCambridge et al., 2003	Goal setting<br><br>Problem solving<br><br>Information about health consequences	No differences in smoking and alcohol outcomes<br><br>No reduction	No differences in smoking and alcohol outcomes<br><br>No reduction	Face to face
Meacham et al., 2021	Problem solving<br><br>Action planning<br><br>Feedback on behaviour<br><br>Information about antecedents<br><br>Prompts/cues<br><br>Pharmacological support	Abstinence from smoking in all groups showed no significant difference<br><br>No reduction	No significant difference in heavy drinking episodes in all groups<br><br>No reduction	Web<br><br>Mail
Pengpid et al., 2015	Feedback on behaviour<br><br>Information about antecedents	Consistent improvements in all outcome measures among study groups<br><br>Reduction	Consistent improvements in all outcome measures among study groups<br><br>Reduction	Face to face

Study	BCTs	Tobacco Outcome	Alcohol Outcome	Mode of Delivery
Ravindranath et al., 2018	Goal setting<br><br>Self-monitoring of behaviour<br><br>Social support	Tobacco and alcohol consumption decreased<br><br>Reduction	Tobacco and alcohol consumption decreased<br><br>Reduction	Face to face
Sabari Sridhar et al., 2017	Feedback on behaviour<br><br>Information about health consequences	Statistically significant reduction in tobacco and alcohol from baseline to follow-up in both groups<br><br>Reduction	Statistically significant reduction in tobacco and alcohol from baseline to follow-up in both groups<br><br>Reduction	Face to face
Sobell et al., 2017	Goal setting<br><br>Problem solving<br><br>Action planning<br><br>Feedback on behaviour<br><br>Information about health consequences<br><br>Pros and cons<br><br>Pharmacological support	Higher smoking cessation rates in intervention group compared to control group<br><br>Reduction	Reduced alcohol consumption in both groups<br><br>Reduction	Face to face<br><br>Over the phone<br><br>Printed Material

Study	BCTs	Tobacco Outcome	Alcohol Outcome	Mode of Delivery
Toll et al., 2015	Goal setting<br><br>Information about health consequences<br><br>Information about antecedents<br><br>Pharmacological support	Intervention group had higher rate of smoking abstinence than control<br><br>Reduction	Reported fewer heavy drinking days than control<br><br>Reduction	Over the phone<br><br>Mail
Vander Weg et al., 2016	Goal setting<br><br>Problem solving<br><br>Action planning<br><br>Pharmacological support	Smoking quit rates higher in the intervention group than in control, but no statistically significant differences in group<br><br>Reduction	Participants who were eligible to participate in the alcohol risk reduction intervention did not agree to participate<br><br>No reduction	Over the phone
Vinci et al., 2023	Goal setting<br><br>Action planning<br><br>Social support<br><br>Pharmacological support	Tobacco use and heavy drinking days were reduced in each group significantly<br><br>Reduction	Tobacco use and heavy drinking days were reduced in each group significantly<br><br>Reduction	App<br><br>Web<br><br>Printed Material
Vrdoljak et al., 2014	Problem solving<br><br>Action planning<br><br>Instruction on how to perform the behaviour	Few smokers stopped smoking<br><br>Reduction	No changes in drinking consumption<br><br>No reduction	Face to face<br><br>Printed Material

Study	BCTs	Tobacco Outcome	Alcohol Outcome	Mode of Delivery
Witkiewitz et al., 2014	Action planning<br><br>Feedback on behaviour<br><br>Information about health consequences<br><br>Behaviour substitution	No differences in smoking and alcohol outcomes<br><br>No reduction	No differences in smoking and alcohol outcomes<br><br>No reduction	Web
