# Supplementary material for: Behaviour change techniques reported in intervention studies of alcohol and tobacco use: a rapid review
Source: Health Psychol Behav Med. 2025 Sep 23;13(1):2554182. doi: 10.1080/21642850.2025.2554182 (PMC12459161; doi:10.1080/21642850.2025.2554182)
Supplement: Appendix A [file RHPB_A_2554182_SM8065.pdf]

Behaviour Change Techniques Reported in Intervention Studies of Alcohol and Tobacco Use: A Rapid Review

Appendix A – Search Strategies

All searches conducted on July 10, 2023

**Ovid MEDLINE: Epub Ahead of Print, In-Process & Other Non-Indexed Citations, Ovid MEDLINE® Daily and Ovid MEDLINE® <1946-Present>**		
1	exp Tobacco Smoking/	6233
2	Tobacco Use Disorder/	12374
3	Smoking Cessation/	32728
4	Tobacco Use Cessation/	1453
5	Smoking Cessation Agents/	253
6	exp "Tobacco Use Cessation Devices"/	2548
7	exp Alcohol Drinking/	78018
8	alcoholism/	80253
9	exp Alcohol-Related Disorders/	121529
10	drinking behavior/	6884
11	alcohol abstinence/	899
12	Combined Modality Therapy/	185059
13	(program* or treat* or intervention* or service* or cessat* or reduc* or quit* or stop* or abstain* or abstin* or withdraw* or recover* or sober or sobriety).ti,kf,hw.	4762998
14	(program* or treat* or intervention* or service* or cessat* or reduc* or quit* or stop* or abstain* or abstin* or withdraw* or recover* or sober or sobriety).ab. /freq=3	3447870
15	(chang* adj3 behav*).ti,ab,kf,hw.	83795
16	or/1-6 [tobacco SHs]	46112
17	or/7-11 [alcohol SHs]	183629
18	or/12-15 [reduction or treatment]	6768150
19	16 and 17 and 18	1468
20	((alcohol* or "alcohol and other drugs" or AOD or heavy drink* or binge drink*) adj3 (concurrent* or comorbid* or co-morbid* or co-occur* or cooccur* or combin* or simultaneous*)).ti,ab,kf,hw.	6480
21	(tobacco* or cigar* or smok* or nicotine).ti,ab,kf,hw. /freq=2	286141
22	Combined Modality Therapy/	185059
23	(program* or treat* or intervention* or service* or cessat* or reduc* or quit* or stop* or abstain* or abstin* or withdraw* or recover* or sober or sobriety or (chang* adj3 behav*)).ti,ab,kf,hw.	12635957
24	20 and 21 and (22 or 23)	517
25	((tobacco* or cigar* or smok* or nicotine) adj3 (concurrent* or comorbid* or co-morbid* or co-occur* or cooccur* or combin* or simultaneous*)).ti,ab,kf,hw.	7518
26	(alcohol* or "alcohol and other drugs" or AOD or heavy drink* or binge drink*).ti,ab,kf,hw. /freq=2	259077
27	Combined Modality Therapy/	185059

28	(program* or treat* or intervention* or service* or cessat* or reduc* or quit* or stop* or abstain* or abstin* or withdraw* or recover* or sober or sobriety or (chang* adj3 behav*)).ti,ab,kf,hw.	12635957
29	25 and 26 and (27 or 28)	592
30	Combined Modality Therapy/	185059
31	treatment outcome/	1152341
32	((concurrent* or comorbid* or co-morbid* or co-occur* or cooccur* or combin* or simultaneous*) adj3 ("use" or user* or used or using or consum* or behav* or disorder* or depend* or addict*)).ti,ab,kf,hw.	256013
33	((concurrent* or comorbid* or co-morbid* or co-occur* or cooccur* or combin* or simultaneous*) adj3 (treat* or intervention* or therap* or program* or service*)).ti,ab,kf,hw.	585581
34	((tobacco* or cigar* or smok* or nicotine) and (alcohol* or heavy drink* or binge drink*)).ti,kf,hw.	26567
35	((tobacco* or cigar* or smok* or nicotine) and (alcohol* or heavy drink* or binge drink*)).ab. /freq=3	14748
36	(30 or 31 or 32 or 33) and (34 or 35)	2204
37	19 or 24 or 29 or 36	3780
38	adolescent/ not adult/	763246
39	37 not 38	3446
40	(Randomized Controlled Trial or Controlled Clinical Trial or Pragmatic Clinical Trial or Clinical Study or Adaptive Clinical Trial or Equivalence Trial).pt.	693604
40	(Randomized Controlled Trial or Controlled Clinical Trial or Pragmatic Clinical Trial or Clinical Study or Adaptive Clinical Trial or Equivalence Trial).pt.	611361
41	(Clinical Trial or Clinical Trial, Phase I or Clinical Trial, Phase II or Clinical Trial, Phase III or Clinical Trial, Phase IV or Clinical Trial Protocol).pt.	335629
42	Multicenter Study.pt.	786
43	Clinical Studies as Topic/	1278013
44	exp Clinical Trial/ or exp Clinical Trials as Topic/ or Clinical Trial Protocol/ or Clinical Trial Protocols as Topic/ or exp "Clinical Trial (topic)"/	355150
45	Multicenter Study/ or Multicenter Studies as Topic/ or "Multicenter Study (topic)"/	106944
46	Randomization/	106944
47	Random Allocation/	175628
48	Double-Blind Method/	175628
49	Double-Blind Studies/	32805
50	Single-Blind Method/	32805
51	Single-Blind Studies/	35930
52	Placebos/	1972

53	Control Groups/	1972
54	Cross-Over Studies/ or Crossover Procedure/	55316
55	(random* or sham or placebo*).ti,ab,hw,kf.	1816274
56	((singl* or doubl*) adj (blind* or dumm* or mask*)).ti,ab,hw,kf.	267729
57	((tripl* or trebl*) adj (blind* or dumm* or mask*)).ti,ab,hw,kf.	1627
58	(control* adj3 (study or studies or trial* or group*)).ti,ab,hw,kf.	1927390
59	(clinical adj3 (study or studies or trial*)).ti,ab,hw,kf.	1434012
60	(Nonrandom* or non random* or non-random* or quasi-random* or quasirandom*).ti,ab,hw,kf.	54723
61	(phase adj3 (study or studies or trial*)).ti,ab,hw,kf.	178336
62	((crossover or cross-over) adj3 (study or studies or trial*)).ti,ab,hw,kf.	76869
63	((multicent* or multi-cent*) adj3 (study or studies or trial*)).ti,ab,hw,kf.	408789
64	allocated.ti,ab,hw.	83984
65	((open label or open-label) adj5 (study or studies or trial*)).ti,ab,hw,kf.	44871
66	((equivalence or superiority or non-inferiority or noninferiority) adj3 (study or studies or trial*)).ti,ab,hw,kf.	12164
67	(pragmatic study or pragmatic studies).ti,ab,hw,kf.	604
68	((pragmatic or practical) adj3 trial*).ti,ab,hw,kf.	7722
69	((quasiexperimental or quasi-experimental) adj3 (study or studies or trial*)).ti,ab,hw,kf.	12275
70	trial.ti,kf.	309512
71	or/40-70	3803916
72	exp animals/	26490480
73	exp models animal/	641092
74	exp animal experiment/	10336
75	exp vertebrate/	25745632
76	72 or 73 or 74 or 75	26492458
77	exp humans/	21353005
78	76 not 77	5139453
79	71 not 78 [CADTH All Clinical Trials Filter]	3404341
80	39 and 79	1020
81	(systematic review or scoping review or meta-analysis or metaanalysis).ti,ab,kf,hw.	455983
82	29 and 81 [reviews for handsearch]	137
83	80 or 82	1079

**Embase Classic+Embase <1947 to 2023 July 07>**		
1	*smoking/	72711
2	*cigarette smoking/	22326
3	*smoking habit/	3050

4	*parental smoking/	370
5	*maternal smoking/	2061
6	*paternal smoking/	94
7	*tobacco dependence/	9698
8	*smoking cessation/	26825
9	*smoking cessation program/	1787
10	exp *alcohol abuse/	12396
11	*drinking behavior/	18179
12	*alcoholism/	87579
13	*alcohol abstinence/	1607
14	*alcohol withdrawal syndrome/	988
15	(program* or treat* or intervention* or service* or cessat* or reduc* or quit* or stop* or abstain* or abstin* or withdraw* or recover* or sober or sobriety).ti,kf,hw.	7612365
16	(program* or treat* or intervention* or service* or cessat* or reduc* or quit* or stop* or abstain* or abstin* or withdraw* or recover* or sober or sobriety).ab. /freq=3	5241710
17	(chang* adj3 behav*).ti,ab,kf,hw.	127394
18	or/1-9 [tobacco SHs]	125331
19	or/10-14 [alcohol SHs]	114147
20	or/15-17 [reduction or treatment]	10286709
21	18 and 19 and 20	1234
22	((alcohol* or "alcohol and other drugs" or AOD or heavy drink* or binge drink*) adj3 (concurrent* or comorbid* or co-morbid* or co-occur* or cooccur* or combin* or simultaneous*)).ti,ab,kf,hw.	10024
23	(tobacco* or cigar* or smok* or nicotine).ti,ab. /freq=2	318152
24	(program* or treat* or intervention* or service* or cessat* or reduc* or quit* or stop* or abstain* or abstin* or withdraw* or recover* or sober or sobriety or (chang* adj3 behav*)).ti,ab,kf,hw. /freq=2	10277582
25	22 and 23 and 24	573
26	((tobacco* or cigar* or smok* or nicotine) adj3 (concurrent* or comorbid* or co-morbid* or co-occur* or cooccur* or combin* or simultaneous*)).ti,ab,kf,hw.	12133
27	(alcohol* or "alcohol and other drugs" or AOD or heavy drink* or binge drink*).ti,ab. /freq=2	269508
28	(program* or treat* or intervention* or service* or cessat* or reduc* or quit* or stop* or abstain* or abstin* or withdraw* or recover* or sober or sobriety or (chang* adj3 behav*)).ti,ab,kf,hw. /freq=2	10277582
29	26 and 27 and 28	602
30	treatment outcome/	958021
31	((concurrent* or comorbid* or co-morbid* or co-occur* or cooccur* or combin* or simultaneous*) adj3 (treat* or intervention* or therap* or program* or service*)).ti,ab,kf,hw.	461895

32	((tobacco* or cigar* or smok* or nicotine) and (alcohol* or heavy drink* or binge drink*)).ti,kf.	11318
33	((tobacco* or cigar* or smok* or nicotine) and (alcohol* or heavy drink* or binge drink*)).ab. /freq=3	20577
34	(30 or 31) and (32 or 33)	590
35	21 or 25 or 29 or 34	2354
36	adolescent/ not adult/	749126
37	35 not 36	2222
38	limit 37 to conference abstracts	469
39	limit 37 to "preprints (unpublished, non-peer reviewed)"	2
40	37 not (38 or 39) [final - all study types]	1751
41	Clinical Studies as Topic/	178232
42	exp Clinical Trial/ or exp Clinical Trials as Topic/ or Clinical Trial Protocol/ or Clinical Trial Protocols as Topic/ or exp "Clinical Trial (topic)"/	2298652
43	Multicenter Study/ or Multicenter Studies as Topic/ or "Multicenter Study (topic)"/	421971
44	Randomization/	99799
45	Random Allocation/	95931
46	Double-Blind Method/	189549
47	Double Blind Procedure/	214408
48	Double-Blind Studies/	171542
49	Single-Blind Method/	50349
50	Single Blind Procedure/	52414
51	Single-Blind Studies/	52414
52	Placebos/	359229
53	Placebo/	415924
54	Control Groups/	123915
55	Control Group/	123915
56	Cross-Over Studies/ or Crossover Procedure/	76075
57	(random* or sham or placebo*).ti,ab,hw,kf.	2593269
58	((singl* or doubl*) adj (blind* or dumm* or mask*)).ti,ab,hw,kf.	369992
59	((tripl* or trebl*) adj (blind* or dumm* or mask*)).ti,ab,hw,kf.	2207
60	(control* adj3 (study or studies or trial* or group*)).ti,ab,hw,kf.	11007122
61	(clinical adj3 (study or studies or trial*)).ti,ab,hw,kf.	6882477
62	(Nonrandom* or non random* or non-random* or quasi-random* or quasirandom*).ti,ab,hw,kf.	71360
63	(phase adj3 (study or studies or trial*)).ti,ab,hw,kf.	398648
64	((crossover or cross-over) adj3 (study or studies or trial*)).ti,ab,hw,kf.	70787

65	((multicent* or multi-cent*) adj3 (study or studies or trial*)).ti,ab,hw,kf.	530280
66	allocated.ti,ab,hw.	110656
67	((open label or open-label) adj5 (study or studies or trial*)).ti,ab,hw,kf.	87845
68	((equivalence or superiority or non-inferiority or noninferiority) adj3 (study or studies or trial*)).ti,ab,hw,kf.	18723
69	(pragmatic study or pragmatic studies).ti,ab,hw,kf.	934
70	((pragmatic or practical) adj3 trial*).ti,ab,hw,kf.	8922
71	((quasiexperimental or quasi-experimental) adj3 (study or studies or trial*)).ti,ab,hw,kf.	19717
72	trial.ti,kf.	454536
73	or/41-72	15031896
74	exp animals/	33012599
75	exp animal experimentation/	3123046
76	exp models animal/	1789754
77	exp animal experiment/	3123046
78	nonhuman/	7532207
79	exp vertebrate/	32126776
80	or/74-79	35113147
81	exp humans/	27022236
82	exp human experiment/	658171
83	81 or 82	27024945
84	80 not 83	8089674
85	73 not 84 [CADTH All Clinical Trials Filter]	11823044
86	40 and 85 [FINAL RESULTS]	1055
87	(systematic review or scoping review or meta-analysis or metaanalysis).ti,ab,kf,hw.	737735
88	40 and 87 [reviews for handsearch]	88
89	86 or 88	1080

**APA PsycInfo <1806 to July Week 1 2023>**		
1	tobacco smoking/	35958
2	tobacco use disorder/	307
3	smoking cessation/	14831
4	nicotine withdrawal/	972
5	exp "alcohol use"/	30440
6	exp alcohol abuse/	51518
7	exp "alcohol use disorder"/	57318
8	drinking behavior/	2102
9	sobriety/	1987
10	exp alcohol treatment/	10062

11	(program* or treat* or intervention* or service* or cessat* or reduc* or quit* or stop* or abstain* or abstin* or withdraw* or recover* or sober or sobriety).ti,id,hw.	952021
12	(program* or treat* or intervention* or service* or cessat* or reduc* or quit* or stop* or abstain* or abstin* or withdraw* or recover* or sober or sobriety).ab. /freq=3	674423
13	(chang* adj3 behav*).ti,ab,id,hw.	59874
14	or/1-4	41396
15	or/5-10	80904
16	or/11-13	1202787
17	14 and 15 and 16	1332
18	((alcohol* or heavy drink* or binge drink*) adj3 (concurrent* or comorbid* or co-morbid* or co-occur* or cooccur* or combin* or simultaneous*)).ti,ab,id,hw.	4075
19	(tobacco* or cigar* or smok* or nicotine).ti,ab,id,hw. /freq=2	60715
20	(program* or treat* or intervention* or service* or cessat* or reduc* or quit* or stop* or abstain* or abstin* or withdraw* or recover* or sober or sobriety or (chang* adj3 behav*)).ti,ab,id,hw.	2104872
21	18 and 19 and 20	303
22	((tobacco* or cigar* or smok* or nicotine) adj3 (concurrent* or comorbid* or co-morbid* or co-occur* or cooccur* or combin* or simultaneous*)).ti,ab,id,hw.	2181
23	(alcohol* or heavy drink* or binge drink*).ti,ab,id,hw. /freq=2	110122
24	(program* or treat* or intervention* or service* or cessat* or reduc* or quit* or stop* or abstain* or abstin* or withdraw* or recover* or sober or sobriety or (chang* adj3 behav*)).ti,ab,id,hw.	2104872
25	22 and 23 and 24	326
26	(therap* or treatment*).hw.	536045
27	((concurrent* or comorbid* or co-morbid* or co-occur* or cooccur* or combin* or simultaneous*) adj3 ("use" or user* or used or using or consum* or behav* or disorder* or depend* or addict*)).ti,ab,id,hw.	57490
28	((concurrent* or comorbid* or co-morbid* or co-occur* or cooccur* or combin* or simultaneous*) adj3 (treat* or intervention* or therap* or program* or service*)).ti,ab,id,hw.	29185
29	((tobacco* or cigar* or smok* or nicotine) and (alcohol* or heavy drink* or binge drink*)).ti.	2756
30	((tobacco* or cigar* or smok* or nicotine) and (alcohol* or heavy drink* or binge drink*)).id,hw.	6406
31	(26 or 27 or 28) and (29 or 30)	1167
32	17 or 21 or 25 or 31	2200
33	(adolescen* not adult*).ti,id,hw.	204943
34	32 not 33	1956

35	exp Clinical Trials/ or exp Clinical Trials as Topic/ or Clinical Trial Protocol/ or Clinical Trial Protocols as Topic/ or exp "Clinical Trial (topic)"/	13632
36	Placebo/	6499
37	Control Group/	959
38	(random* or sham or placebo*).ti,ab,hw,id.	273955
39	((singl* or doubl*) adj (blind* or dumm* or mask*)).ti,ab,hw,id.	28901
40	((tripl* or trebl*) adj (blind* or dumm* or mask*)).ti,ab,hw,id.	117
41	(control* adj3 (study or studies or trial* or group*)).ti,ab,hw,id.	197018
42	(clinical adj3 (study or studies or trial*)).ti,ab,hw,id.	74079
43	(Nonrandom* or non random* or non-random* or quasi-random* or quasirandom*).ti,ab,hw,id.	6417
44	(phase adj3 (study or studies or trial*)).ti,ab,hw,id.	9248
45	((crossover or cross-over) adj3 (study or studies or trial*)).ti,ab,hw,id.	5287
46	((multicent* or multi-cent*) adj3 (study or studies or trial*)).ti,ab,hw,id.	9427
47	allocated.ti,ab,hw.	12871
48	((open label or open-label) adj5 (study or studies or trial*)).ti,ab,hw,id.	5214
49	((equivalence or superiority or non-inferiority or noninferiority) adj3 (study or studies or trial*)).ti,ab,hw,id.	1182
50	(pragmatic study or pragmatic studies).ti,ab,hw,id.	144
51	((pragmatic or practical) adj3 trial*).ti,ab,hw,id.	1112
52	((quasiexperimental or quasi-experimental) adj3 (study or studies or trial*)).ti,ab,hw,id.	7022
53	trial.ti,id.	43512
54	or/35-53	461151
55	exp animals/	376140
56	exp animal experiment/	646
57	exp vertebrate/	344251
58	animal.po.	438761
59	55 or 56 or 57 or 58	453000
60	human.po.	4567718
61	59 not 60	390071
62	54 not 61 [CADTH filter]	429393
63	34 and 62	427
64	(systematic review or scoping review or meta-analysis or metaanalysis).ti,ab,id,hw.	76861
65	34 and 64	69
66	63 or 65	462

Cumulative Index to Nursing and Allied Health Literature (CINAHL)		
S1	(MM "Alcohol-Related Disorders+")	29,816
S2	(MJ "Smoking") OR (MJ "Smoking Cessation")	47,864
S3	(MJ "Nicotine Replacement Therapy") OR (MJ "Nicotine Withdrawal") OR (MJ "Tobacco Use Cessation Products")	906
S4	(MM "Alcohol-Related Disorders+")	29,816
S5	(MM "Drinking Behavior+")	22,903
S6	(MM "Alcohol Rehabilitation Programs+")	1,656
S7	TI ((program* or treat* or intervention* or service* or cessat* or reduc* or quit* or stop* or abstain* or abstin* or withdraw* or recover* or sober or sobriety)) OR MJ ((program* or treat* or intervention* or service* or cessat* or reduc* or quit* or stop* or abstain* or abstin* or withdraw* or recover* or sober or sobriety))	1,304,029
S8	(chang* n3 behav*)	40,330
S9	S2 OR S3	48,023
S10	S4 OR S5 OR S6	47,516
S11	S7 OR S8	1,330,255
S12	S9 AND S10 AND S11	496
S13	((alcohol* or heavy drink* or binge drink*) N3 (concurrent* or comorbid* or co-morbid* or co-occur* or cooccur* or combin* or simultaneous*))	3,103
S14	TI (tobacco* or cigar* or smok* or nicotine) OR MJ (tobacco* or cigar* or smok* or nicotine)	72,479
S15	(program* or treat* or intervention* or service* or cessat* or reduc* or quit* or stop* or abstain* or abstin* or withdraw* or recover* or sober or sobriety or (chang* N3 behav*))	3,221,970
S16	S13 AND S14 AND S15	201
S17	((tobacco* or cigar* or smok* or nicotine) N3 (concurrent* or comorbid* or co-morbid* or co-occur* or cooccur* or combin* or simultaneous*))	3,514
S18	TI (alcohol* or heavy drink* or binge drink*) OR MJ (alcohol* or heavy drink* or binge drink*)	68,780
S19	(program* or treat* or intervention* or service* or cessat* or reduc* or quit* or stop* or abstain* or abstin* or withdraw* or recover* or sober or sobriety or (chang* N3 behav*))	3,221,970
S20	S17 AND S18 AND S19	230
S21	MJ ((therap* or treatment*))	1,311,288
S22	((concurrent* or comorbid* or co-morbid* or co-occur* or cooccur* or combin* or simultaneous*) N3 ("use" or user* or used or using or consum* or behav* or disorder* or depend* or addict*))	83,313
S23	TI (((tobacco* or cigar* or smok* or nicotine) and (alcohol* or heavy drink* or binge drink*)))	2,767
S24	MJ (((tobacco* or cigar* or smok* or nicotine) and (alcohol* or heavy drink* or binge drink*)))	2,982
S25	(S21 or S22) AND (S23 OR S24)	538

S26	S12 OR S16 OR S20 OR S25	1,025
S27	TI ((adolescen* not adult*)) OR MJ ((adolescen* not adult*))	160,688
S28	S26 not S27	886
S29	(((MH "Experimental Studies+") OR (MH "Multicenter Studies") OR (MH "Random Sample+") OR (MH "Placebos") OR (MH "Control (Research)+") OR (MH "Crossover Design") OR ((TI random* OR AB random*) OR (TI sham OR AB sham) OR (TI placebo* OR AB placebo*)) OR (((TI singl* OR AB singl*) OR (TI doubl* OR AB doubl*)) W1 ((TI blind* OR AB blind*) OR (TI dumm* OR AB dumm*) OR (TI mask* OR AB mask*))) OR (((TI tripl* OR AB tripl*) OR (TI trebl* OR AB trebl*)) W1 ((TI blind* OR AB blind*) OR (TI dumm* OR AB dumm*) OR (TI mask* OR AB mask*))) OR ((TI control* OR AB control*) N3 ((TI study OR AB study) OR (TI studies OR AB studies) OR (TI trial* OR AB trial*) OR (TI group* OR AB group*))) OR ((TI clinical OR AB clinical) N3 ((TI study OR AB study) OR (TI studies OR AB studies) OR (TI trial* OR AB trial*))) OR ((TI Nonrandom* OR AB Nonrandom*) OR (TI "non random*" OR AB "non random*") OR (TI "non-random*" OR AB "non-random*") OR (TI "quasi-random*" OR AB "quasi-random*") OR (TI quasirandom* OR AB quasirandom*)) OR ((TI phase OR AB phase) N3 ((TI study OR AB study) OR (TI studies OR AB studies) OR (TI trial* OR AB trial*))) OR (((TI crossover OR AB crossover) OR (TI "cross-over" OR AB "cross-over")) N3 ((TI study OR AB study) OR (TI studies OR AB studies) OR (TI trial* OR AB trial*))) OR (((TI multicent* OR AB multicent*) OR (TI "multi-cent*" OR AB "multi-cent*")) N3 ((TI study OR AB study) OR (TI studies OR AB studies) OR (TI trial* OR AB trial*))) OR (TI allocated OR AB allocated) OR (((TI "open label" OR AB "open label") OR (TI "open-label" OR AB "open-label")) N5 ((TI study OR AB study) OR (TI studies OR AB studies) OR (TI trial* OR AB trial*))) OR (((TI equivalence OR AB equivalence) OR (TI superiority OR AB superiority) OR (TI "non-inferiority" OR AB "non-inferiority") OR (TI noninferiority OR AB noninferiority)) N3 ((TI study OR AB study) OR (TI studies OR AB studies) OR (TI trial* OR AB trial*))) OR ((TI "pragmatic study" OR AB "pragmatic study") OR (TI "pragmatic studies" OR AB "pragmatic studies")) OR (((TI pragmatic OR AB pragmatic) OR (TI practical OR AB practical)) N3 (TI trial* OR AB trial*)) OR (((TI quasiexperimental OR AB quasiexperimental) OR (TI "quasi-experimental" OR AB "quasi-experimental")) N3 ((TI study OR AB study) OR (TI studies OR AB studies) OR (TI trial* OR AB trial*))) OR TI trial)	1,278,105
S30	S28 AND S29	257
S31	(systematic review or scoping review or meta-analysis or metaanalysis)	273,835
S32	S28 AND S31	40
S33	S30 OR S32	276
